# Synergy of retinoic acid and BH3 mimetics in *MYC(N)*-driven embryonal nervous system tumours

**DOI:** 10.1038/s41416-024-02740-5

**Published:** 2024-06-28

**Authors:** Till Seiboldt, Constantia Zeiser, Duy Nguyen, Simay Celikyürekli, Sonja Herter, Sara Najafi, Alexandra Stroh-Dege, Chris Meulenbroeks, Norman Mack, Rabia Salem-Altintas, Frank Westermann, Matthias Schlesner, Till Milde, Marcel Kool, Tim Holland-Letz, Meike Vogler, Heike Peterziel, Olaf Witt, Ina Oehme

**Affiliations:** 1 Hopp Children’s Cancer Center Heidelberg (KiTZ), Heidelberg, Germany; 2 Clinical Cooperation Unit Pediatric Oncology (B310), German Cancer Research Center (DKFZ) and German Cancer Consortium (DKTK), Heidelberg, Germany; 3 National Center for Tumor Diseases Heidelberg, Heidelberg, Germany; 4 Faculty of Medicine, Heidelberg University, Heidelberg, Germany; 5 Bioinformatics and Omics Data Analytics, German Cancer Research Center (DKFZ), Heidelberg, Germany; 6 Faculty of Biosciences, Heidelberg University, Heidelberg, Germany; 7 Department of Pediatric Oncology, Hematology, Immunology and Pulmonology, Heidelberg University Hospital, Heidelberg, Germany; 8 Princess Máxima Center for Pediatric Oncology, Utrecht, The Netherlands; 9 Division of Pediatric Neurooncology, German Cancer Research Center (DKFZ) and German Cancer Consortium (DKTK), Heidelberg, Germany; 10 Division of Neuroblastoma Genomics, German Cancer Research Center (DKFZ), Heidelberg, Germany; 11 Biomedical Informatics, Data Mining and Data Analytics, Faculty of Applied Computer Science and Medical Faculty, University of Augsburg, Augsburg, Germany; 12 University Medical Center Utrecht, Utrecht, the Netherlands; 13 Division of Biostatistics, German Cancer Research Center (DKFZ), Heidelberg, Germany; 14 Institute for Experimental Pediatric Hematology and Oncology, Goethe-University Frankfurt, Frankfurt, Germany; 15 German Cancer Consortium (DKTK) partner site Frankfurt/Mainz, a partnership between DKFZ and University Hospital Frankfurt, Frankfurt, Germany; 16 University Cancer Center Frankfurt (UCT), University Hospital Frankfurt, Goethe-University Frankfurt, Frankfurt, Germany

**Keywords:** Embryonal neoplasms, Targeted therapies

## Abstract

**Background:**

Certain paediatric nervous system malignancies have dismal prognoses. Retinoic acid (RA) is used in neuroblastoma treatment, and preclinical data indicate potential benefit in selected paediatric brain tumour entities. However, limited single-agent efficacy necessitates combination treatment approaches.

**Methods:**

We performed drug sensitivity profiling of 76 clinically relevant drugs in combination with RA in 16 models (including patient-derived tumouroids) of the most common paediatric nervous system tumours. Drug responses were assessed by viability assays, high-content imaging, and apoptosis assays and RA relevant pathways by RNAseq from treated models and patient samples obtained through the precision oncology programme INFORM (*n* = 2288). Immunoprecipitation detected BCL-2 family interactions, and zebrafish embryo xenografts were used for in vivo efficacy testing.

**Results:**

Group 3 medulloblastoma (MB_G3_) and neuroblastoma models were highly sensitive to RA treatment. RA induced differentiation and regulated apoptotic genes. RNAseq analysis revealed high expression of *BCL2L1* in MB_G3_ and *BCL2* in neuroblastomas. Co-treatments with RA and BCL-2/X_L_ inhibitor navitoclax synergistically decreased viability at clinically achievable concentrations. The combination of RA with navitoclax disrupted the binding of BIM to BCL-X_L_ in MB_G3_ and to BCL-2 in neuroblastoma, inducing apoptosis in vitro and in vivo.

**Conclusions:**

RA treatment primes MB_G3_ and NB cells for apoptosis, triggered by navitoclax cotreatment.

## Introduction

Although past decades have seen a remarkable increase in childhood cancer survival to above 80%, it remains the leading cause of death from disease in children beyond infancy [1]. Particularly relapsed tumours of the peripheral and central nervous system, still carry dismal prognoses and significantly contribute to childhood cancer mortality [1, 2]. In the case of tumour recurrence, few therapeutic options remain with precision oncology approaches, such as the INFORM (INdividualized Therapy FOr Relapsed Malignancies in Childhood) programme, identifying survival-prolonging actionable targets in just up to 10% of cases [3]. Importantly, disease- and treatment-related morbidities, such as chronic cardiovascular disease, secondary malignancy, and neurological impairment, also burden the lives of many childhood cancer survivors, including patients treated for CNS malignancies [4].

The most common diagnoses of CNS malignancies in children are medulloblastoma (MB), high-grade glioma (HGG) and ependymoma (EPN). To accurately and reliably differentiate between the numerous heterogeneous subtypes of childhood brain tumours, molecular analyses of the genome and methylome are now routinely incorporated into diagnostics [5]. In MB, four distinct molecular subgroups have been identified, with Group 3 MB (MB_G3_) patients having the worst prognosis [6, 7]. Despite surgical resection, craniospinal irradiation, and intensive chemotherapy, the 5-year overall survival is less than 50% in these high-risk MB cases [7, 8]. Exceptionally poor survival can be observed in the diverse spectrum of paediatric HGG, where molecularly informed classification is based on histone 3 (H3) mutation status [9, 10]. In EPN, the two particularly aggressive subgroups, supratentorial ZFTA fusion-positive and posterior fossa group A (PFA) EPN, mostly occur in childhood [10, 11].

The most common extracranial tumour in childhood, neuroblastoma (NB), originates during embryogenesis from blocked differentiation of neural crest-derived precursor cells of the developing sympathetic nervous system [12]. It has a large spectrum of disease courses, ranging from spontaneous regression and maturation to aggressively progressing disease despite intensive multimodal treatment [13]. Risk-adapted treatment may only consist of observation or surgical resection and chemotherapy in low- to intermediate-risk cases [14]. In high-risk NB, which is often driven by amplification of the *MYCN* oncogene (NB_MYCN_), however, intensive chemotherapy alongside surgery, myeloablation and autologous stem-cell transplantation, radiation, and maintenance therapy with anti-GD2 immunotherapy and retinoic acid (RA) only achieves a 5-year overall survival of approximately 60% [13, 15]. As first-line therapeutic in high-risk NB care, RA is a component of maintenance therapy in current treatment recommendations from the North American Children’s Oncology Group and the International Society of Paediatric Oncology Europe Neuroblastoma Group [15–17]. The standard paediatric treatment regimen for high-risk NB with 13-*cis*-RA leads to serum concentrations in the low micromolar range (trough to peak: 4.1–7.2 µM) and is well tolerated by patients [18]. Furthermore, 13-*cis*-RA permeates the blood-brain barrier (BBB) well, with peak white matter concentrations reaching approximately half of the peak serum concentrations in a preclinical rat model [19]. Additionally, it has demonstrated antiproliferative, differentiating, and proapoptotic effects against preclinical models of multiple other paediatric nervous system tumour entities [20–24].

To identify novel treatment approaches, mechanisms by which relapsed paediatric nervous system tumours evade current treatment approaches, are of particular interest. The B-cell lymphoma 2 (BCL-2) family of proteins plays an important role in maintaining homoeostasis in apoptosis by regulating mitochondrial outer membrane permeabilization (MOMP) through the interaction of its pro- and anti-apoptotic members [25]. Importantly, the main anti-apoptotic proteins BCL-2, B-cell lymphoma-extra large (BCL-X_L_) and myeloid cell leukaemia 1 (MCL-1) are often upregulated across many adult and paediatric cancer types [26]. To address this mechanism, small-molecule BCL-2 homology 3 (BH3) mimetics, such as navitoclax and venetoclax, bind to the hydrophobic groove of antiapoptotic and pore-forming BCL-2 proteins, thus antagonising BCL-2 and BCL-X_L_ and inducing MOMP [25, 27]. These therapeutics display compelling strategies to target cancer cells and have been under intensive clinical investigation in a variety of cancers as single agents and combined with other therapies [25].

As many subtypes of the diverse spectrum of paediatric nervous system cancers have high rates of aggressive progression or relapse and current standard-of-care approaches result in severe chronic health conditions, novel targeted treatment strategies need to be explored to potentially improve efficacy and reduce toxicity. Here, we investigated a molecularly diverse panel of high-risk paediatric nervous system tumours for their RA response as a single drug and in combination with a library of 76 clinically relevant compounds, with the aim of identifying improved combination therapies for this unmet clinical need.

## Materials and methods

### Cell culture

Cell culture was performed as described previously [28]. The *N* = 16 culture models applied for the drug screen included, besides established cell lines, the patient-derived cultures NB-S-124 (NB), SU-DIPG-13 (HGG) and SU-DIPG-25 (HGG) (both kindly provided by Michelle Monje, Stanford, CA, USA), and INF_R_1073_relapse1_LTC (HGG) and INF_R_859_primary_LTC (HGG) (both derived from primary INFORM tumour samples; LTC: longterm culture). Further validation experiments were performed using the patient-derived tumouroid cultures INF_R_1632_relapse1_PDX_LTC (NB; from mouse PDX), INF_R_1887_relapse1_LTC (EPN), Med_2112fh_PDX_FTC (MB_G3_; from mouse PDX; FTC: fresh tissue culture), and RCMB28 (MB_G3_). INFORM long-term cultures (LTC) were established from primary tumour samples from the INFORM programme as previously described [3, 28, 29]. All patient-derived models were cultured organoid-like as free-floating and semiadherent spheroids under serum-free conditions in stem-cell medium. The Supplementary Information gives a detailed description about methods and models.

### Drugs and reagents

13-*cis*-RA (MedChem Express, Monmouth Junction, NJ, USA), A-1155463 (Selleckchem, Houston, TX, USA), all-*trans*-RA (Selleckchem), entinostat (Biomol GmbH, Hamburg, Germany), I-BET151 (Selleckchem), navitoclax (TargetMol, Wellesley Hills, MA, USA), staurosporine (Selleckchem), and venetoclax (Selleckchem) were dissolved in dimethyl sulfoxide (DMSO, Sigma-Aldrich) to a 10 mM stock concentration and stored as aliquots at −80 °C protected from light.

### Metabolic activity assays and medium-throughput drug combination screening

The assays were performed as described previously [28, 30, 31]. All culture models were seeded as three-dimensional tumouroids on round-bottom 384-well ultralow attachment plates (Corning Inc., Corning, NY, USA). The Supplementary Information gives a detailed description.

### Bulk RNA sequencing

For bulk RNA sequencing, two medulloblastoma (MB) (D425, HD-MB03) and two neuroblastoma (NB) (NB-S-124, SK-N-BE(2)-C) cultures were chosen. Cells were seeded on 10 cm dishes, incubated for 24 h and subsequently treated for 144 h with 0.5/1 µM (MB/NB) all-*trans*-RA or DMSO solvent control in two independent replicate experiments. Total RNA was extracted using the RNeasy Mini Kit (Qiagen, Hilden, Germany) according to the manufacturer’s protocol. RNA integrity and quantity were assessed with a 2100 Bioanalyzer (Agilent Technologies, Santa Clara, CA, USA). After passing initial quality control at the in-house DKFZ Genomics & Proteomics Core Facility, RNAseq was performed on Illumina HiSeq 4000 Single-read 50 bp (Illumina, San Diego, CA, USA) using the TruSeq stranded Protocol. Read quality control was passed with Q30 > 97.5% in all samples. Read mapping and counting were carried out by the RSEM pipeline using the Bowtie2 alignment programme and Expectation Maximization (EM) algorithm for gene-level read estimates [32, 33]. The data is available on the online platform R2 (http://r2.amc.nl): Exp Medulloblastoma / Neuroblastoma RA - Oehme - 20 - rsem - gencode19. Subsequent differential expression analysis was performed using the DESeq2 package [34] in R. Gene Ontology (GO) analysis was performed using clusterProfiler package [35].

### Synergy analysis

For synergy analysis, a 5 × 5 matrix and seven ray design of RA combined with entinostat, I-BET151 or navitoclax was used. Cells were seeded as three-dimensional tumouroids on round-bottom 384-well plates and treated for 144 h, and readout was performed as described in section “Metabolic activity assays and medium-throughput drug combination screening”. To assess combination effects, the experimentally measured antitumour effect was compared to the expected response determined using the Loewe reference model, as independent drug effects cannot be assumed in these combinations. Synergy scores were calculated and visualised with SynergyFinder web-application 2.0 (https://synergyfinder.fimm.fi) [36] for analysis of the matrix and a custom R pipeline for ray designs based on the DRC R-package [37] (sample R script included in supplementary information). Loewe Combination Indices were calculated according to Lee et al [38].

### Colony formation assay

To assess clonogenic growth and sustained treatment effect after drug removal, cells were seeded on six-well plates at 2000 cells per well and treated after 24 h for 96 h. Then, drugs were removed by medium change and cells were cultured for another 7 d before undergoing fixation and staining with 1% crystal violet solution. Colonies per well were quantified using the ITCN plugin in ImageJ Fiji version 2.5.0.

### Morphology assays

To observe morphological effects upon prolonged treatment, cells were seeded on six-well plates (100,000 cells/well HD-MB03; 20,000 cells/well SK-N-BE(2)-C; 10,000 cells/well SJ-GBM2) and treated the next day for additional 8 d. Fixation and staining was performed with 1% crystal violet solution. Three random sites per replicate were captured with light microscopy (Nikon Eclipse TS2 10x magnification).

### High-content fluorescence microscopy (HCM)

For HCM, cells were seeded as a monolayer on a black high-content imaging ½ area 96-well microplate with a flat glass bottom (Corning Inc.). To achieve monolayer attachment for NB-S-124 cells, corresponding wells underwent surface coating with 1:100 diluted Matrigel (Corning Inc.) before seeding. Treatment was carried out the day after seeding using the TECAN D300e drug dispenser. After 72 h of treatment, cells were stained with fluorescence dyes for 30 min as indicated in Supplementary Table 1. Live cell imaging was performed with an ImageXpress Micro Confocal High-content microscope (Molecular Devices, San José, CA, USA) at 10x magnification. CellProfiler Version 4.2.5 [39] and CellProfiler Analyst Version 3.0.4 [40] was used for automated image analysis. To quantify the number of Caspase positive cells and fragmented nuclei, a custom CellProfiler pipeline and the CellProfiler Analyst machine-learning tool Classifier was used as described in Supplementary Table 2. To quantify the length of neurite-like protrusions, a custom CellProfiler pipeline was used as described in Supplementary Table 3. The high-content imaging of patient-derived tumouroids for validation was performed as described in the Supplementary Information.

### Caspase-3/7 activity assay

To measure caspase-3/7 activity, the Caspase-3/7 Fluorometric Assay (BioVision, Abcam, Cambridge, UK) was performed following the manufacturer’s instructions and as described previously [41]. Details are listed in the Supplementary Information.

### Trypan blue assays

To detect cell viability and cell death, trypan blue assays were performed as described previously [41]. Details are listed int the Supplementary Information.

### Western blot analysis and immunoprecipitation

Protein expression analysis was performed as reported previously [41]. The immunoprecipitation method was described previously [42]. Details are listed in Supplementary Information.

### Zebrafish lines, toxicity assays, embryo xenotransplantation, and treatment

In vivo zebrafish (*Danio rerio*) embryo xenograft was performed as previously described [43]. Details are listed in the Supplementary Information.

### Patient-derived tumour xenotransplantation

Mouse PDX were performed as previously described [44]. Details are listed in the Supplementary Information.

### Statistical analysis

If not indicated otherwise, experiments were carried out as independent triplicates. GraphPad Prism 5 software (Version 5.01, GraphPad Software, San Diego, CA, USA) was used to calculate dose-response curves and corresponding absolute half-maximal inhibiting concentrations (IC_50_). RStudio (Version 2022.07.1 + 554, RStudio PBC, Boston, MA, USA) was used for statistical analysis and plotting with R (Version 4.2.1, R Foundation for Statistical Computing, Vienna, Austria) and R packages tidyr, dplyr and ggplot2. To compare data, a two-tailed unpaired *t*-test or, where appropriate, one-way analysis of variance (ANOVA) with Tukey’s post hoc corrections were applied. *p* < 0.05 were considered significant (*p < 0.05, **p < 0.01, ***p < 0.001).

### Gene expression analysis R2

Web-based gene expression analysis R2 (R2: microarray analysis and visualisation platform; http://r2.amc.nl) was used to investigate the expression of regulated genes in the MB and NB cell models after RA treatment (Medulloblastoma / Neuroblastoma RA - Oehme - 20 - rsem - gencode19; 2021-12-01). Further R2 based gene expression analyses were performed using the *Brain Tumor (2023-01-23) - Kool - 123 - MAS5.0 - u133p2 data set produced by Marcel Kool* and *Neuroblastoma - Westermann - 39 - TMM - ensh37e75 data set produced by Frank Westermann* and in the INFORM 2288 patient cohort (2023-07-05), produced and kindly provided by the INFORM programme [3, 29, 31, 45].

## Results

### High-risk neuroblastoma and Group 3 medulloblastoma models share RA sensitivity

**Fig. 1:**
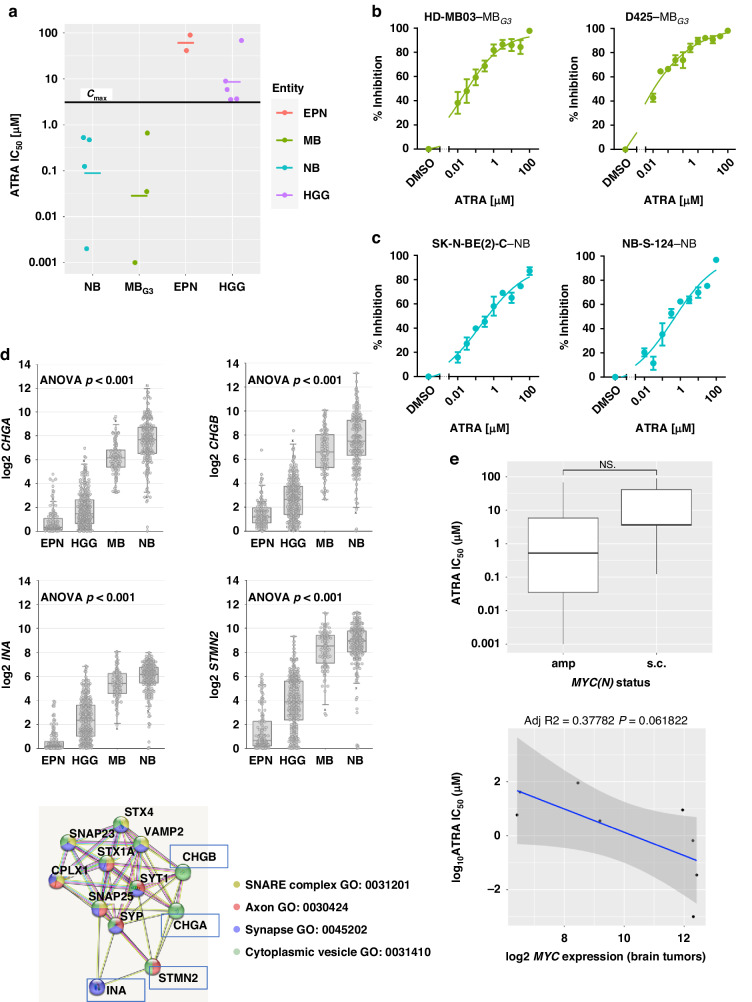
Neuroblastoma and Group 3 medulloblastoma cells share antitumoural response patterns to retinoic acid treatment. **a** Retinoic acid sensitivity as measured after 144 h of ATRA treatment through the metabolic activity CellTiter Glo assay. Absolute IC_50_ values of *N* = 14 culture models (including the primary patient-derived cultures NB-S-124, SU-DIPG-13, SU-DIPG-25, INF_R_1073_relapse1_LTC, and INF_R_859_primary_LTC) depicted separated by entity and compared to the estimated ATRA white matter C_max_ (3.1 µM). All cells were seeded as three-dimensional tumouroids. The bar indicates the corresponding mean. In two out of *N* = 16 tested culture models RA IC_50_ values could not be calculated (>100 µM) due to no dose-dependent response (ONS-76, SU-DIPG-13). **b**, **c** ATRA dose-response curves of selected MB_G3_ and NB cultures after 144 h. The % Inhibition was calculated using the raw values of the metabolic activity at a respective drug concentration compared to the positive and negative controls. Error bars indicate standard deviation. *N* = 3 **d** Dot box plots of genes that are significantly upregulated in both relapsed neuroblastoma and medulloblastoma cases compared to ependymoma and high-grade glioma from the INFORM programme as measured by RNA sequencing (log2-fold-change expression). EPN, *N* = 128; HGG, *N* = 298; MB, *N* = 103; NB, *N* = 223. Connecting GO terms identified from the STRING protein interaction analysis of the four shared significantly upregulated genes. **e** Box plot of absolute RA IC_50_ value of *N* = 14 culture models compared to known genomic amplification status of either *MYC/MYCN* or single copy variant, and dot plot depicting absolute RA IC_50_ values and microarray-derived log_2_ *MYC* expression of 8 brain tumour culture models with linear regression analysis. Out of the *N* = 10 brain tumour culture models with measurable RA IC_50_ values two were excluded due to missing expression data (SJ-GBM-2, KNS-42). Amp amplified, ANOVA analysis of variance, ATRA all-*trans*-retinoic acid, C_max_ peak concentration, EPN ependymoma, GO gene ontology, HGG high-grade glioma, IC_50_ half-maximal inhibitory concentration, MB medulloblastoma, MB_G3_ Group 3 medulloblastoma, NB neuroblastoma, s.c. single copy.

To assess the retinoic acid (RA) sensitivity of a diverse range of hard-to-treat paediatric nervous system tumours, a panel of 16 models (cell lines and ex vivo patient-derived models) was selected for investigation. The panel comprised four neuroblastoma (NB), four medulloblastoma (MB), two ependymoma (EPN) and six paediatric high-grade glioma (HGG) models reflecting different high-risk subgroups, molecular characteristics and growth kinetics (Fig. 1a, Supplementary Table S4). Testing for all models was performed uniformly on three-dimensional spheroids in round-bottom plates to better reflect the in vivo conditions with growth patterns, metabolism, cell-cell contacts, and treatment resistance [31, 46]. Upon 144 h of treatment with the single agent all-*trans-*RA (ATRA), tumour cells responded differently depending on the (sub)entity. High-risk NB and *MYC*-amplified MB_G3_ models demonstrate good RA sensitivity with absolute half-maximal inhibitory concentrations (IC_50_ values) well below the peak serum concentration (C_max_) for the standard dosing regimen in paediatric oncology [18] (Fig. 1a–c, Supplementary Table S4). The most pronounced antitumoural activity was observed in CHP134 (NB_MYCN_, IC50 = 2 nM), D425 (MB_G3_, IC50 = 1 nM), and HD-MB03 (MB_G3_, IC_50_ = 35 nM) (Fig. 1b, c). The one model for Sonic hedgehog MB (MB_SHH_), and the models for EPN subgroups (ZFTA and PFA), and HGG subgroups (K27M, G34V/R and wildtype), however, showed little to no RA response at clinically relevant concentrations (Supplementary Table S4). Thus, we defined RA responsive models as those achieving at least 50% inhibition at concentrations below the C_max_ (7.2 µM for NB, 3.1 µM for CNS tumours as levels are half as high beyond the blood-brain barrier [19]), which includes all NB and all MB_G3_ models, while the remaining nine models were classified as RA less-responsive. To capture common features of the responsive entities MB and NB versus differences to the less-responsive entities HGG and EPN, we used the INFORM patients gene expression data set of mainly relapsed tumours on the online platform R2 (Genomics Analysis and Visualisation Platform (http://r2.amc.nl)) and identified the top 50 differentially regulated genes (TOP50) between two groups for the comparison MB versus HGG, MB versus EPN, NB versus HGG and NB versus EPN (Supplementary Tables 5–8). From all four TOP50 gene lists, only four genes overlapped, which were significantly upregulated in NB and MB (especially in G3/G4) for all comparisons, namely *CHGA*, *CHGB*, *INA* and *STMN2* (Fig. 1d; Supplementary Fig. S1). The protein interaction tool STRING (string-db.org) [47] identified GO terms relevant to synaptic secretory vesicles as a connecting theme (Fig. 1d). While the six *MYC(N)*-amplified MB and NB cell lines plus high-*MYC-*expressing SH-SY5Y cells showed the highest RA sensitivity of all models, it is important to note that *MYC(N)* amplification is not a suitable biomarker of RA sensitivity, as this feature was also found in less-responsive models (Fig. 1e; Supplementary Fig. S2). Hence, ATRA responsiveness appears not to be restricted to *MYC(N)* amplification nor growth kinetics in a given tumour model but rather favours embryonal tumours of the nervous system with synaptic vesicle formation capacity.

### Combination drug screening identifies BCL-X_L_ inhibitors (A-1155463, navitoclax) as top hits across RA-sensitive models

**Fig. 2:**
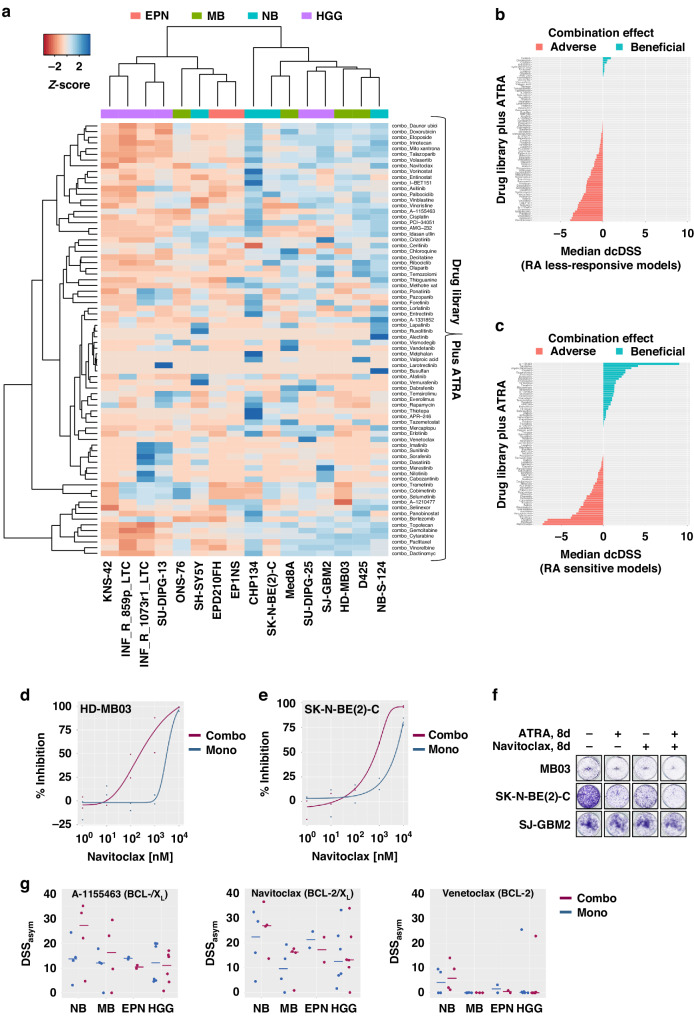
Retinoic acid combination drug library screening reveals beneficial combination partners. **a** Hierarchical clustering analysis of modified drug sensitivity scores for 76 clinically relevant drugs in combination with retinoic acid in *N* = 16 culture models. Treatment on three-dimensional tumouroids was performed for 144 h with the addition of a fixed RA concentration or solvent control (DMSO). **b**, **c** Median dcDSS values of all 76 library drugs for *N* = 9 RA less-responsive models and *N* = 7 RA sensitive models, respectively. Positive dcDSS values indicate beneficial (blue) and negative values indicate adverse (blue) combination effects. **d**, **e** Navitoclax dose-response curves navitoclax with (combo) or without (mono) the fixed RA concentration in cell lines HD-MB03 and SK-N-BE(2)-C. **f** Representative longterm growth assay wells of HD-MB03, SK-N-BE(2)-C and SJ-GBM2 cells treated as indicated for 8 days. **g** Dotplots of DSS_asym_ values for BH3 mimetics A-1155463 (selective BCL-X_L_-inhibitor), navitoclax (BCL-2/X_L_-inhibitor), and venetoclax (selective BCL-2-inhibitor) with (combo) or without (mono) the fixed RA concentration in *N* = 16 culture models separated by entity (NB, *N* = 4; MB, *N* = 4; EPN, *N* = 2; HGG, *N* = 6). The bar indicates the corresponding median DSS_asym_. DSS_asym_ modified drug sensitivity score, dcDSS differential combination DSS, RA retinoic acid.

To identify beneficial combination treatments to either significantly enhance RA treatment in sensitive tumours or overcome RA resistance, a medium-throughput drug combination screen was performed on the above-described panel of 16 models. To this end, we combined the KiTZ drug library of 76 clinically relevant compounds, most of which are already approved or in late-stage clinical trials [31], with a fixed ATRA concentration (1 µM in NB, 0.5 µM in CNS tumours) or solvent control and treated cells for 144 h. The modified drug sensitivity score (DSS_asym_) was used to assess the respective treatment efficacy, and the differential combination DSS (dcDSS) was used to detect possible beneficial or adverse effects of drug combinations [30]. Hierarchical cluster analysis of DSS_asym_ (Combo) values revealed two response pattern groups of eight models each, with the six *MYC(N)-*amplified MB and NB models clustering together (Fig. 2a). Selecting effective combinations by dcDSS (>2) and DSS_asym_ (>10) for treatment efficacy, no overall beneficial combination was identified in RA less-responsive models (highest median dcDSS_Ceritinib_ = 0.95) with mostly negative dcDSS values (Fig. 2b, Supplementary Fig. S3). In RA-responsive models, the BCL-2 family inhibitor navitoclax was identified as top hit (median dcDSS 4.1) (Fig. 2c–g, Supplementary Fig. S4) and confirmed by longterm growth assay (Fig. 2f). The selective BCL-X_L_ inhibitor A-1155463 also classified as top hit (median dcDSS 9.1) (Fig. 2g, Supplementary Fig. S4). Other apoptotic modulators, such as the selective BCL-2 inhibitor venetoclax (median dcDSS 1.2) and MCL-1 inhibitor A-1210477 (median dcDSS 0.0), showed no consistent combination benefit across different models (Fig. 2g, Supplementary Fig. S4). When entities were analysed separately, entinostat (class I HDAC inhibitor) and I-BET151 (BET bromodomain inhibitor) displayed entity-specific combination benefits with RA in MB and NB, respectively (Supplementary Figs. S5–6). In the two EPN (highest median dcDSS_Rapamycin_ = 2.9, DSS_asym_ < 5) and six HGG (highest median dcDSS_Selumetinib_ = 1.9) models, no entity-specific combination partner for RA treatment was identified. In these RA less-responsive models, the combination with RA had a rather adverse effect on the treatment efficacy of most drugs (Fig. 2b, Supplementary Fig. S7–8). In RA-sensitive models, the least favourable combination partners for RA treatment were the antimetabolite methotrexate and the conventional chemotherapeutics paclitaxel, topotecan, mercaptopurine, vincristine, and gemcitabine (Fig. 2c).

### RA/navitoclax cotreatment synergistically inhibits *MYC(N)*-amplified MB and NB proliferation

**Fig. 3:**
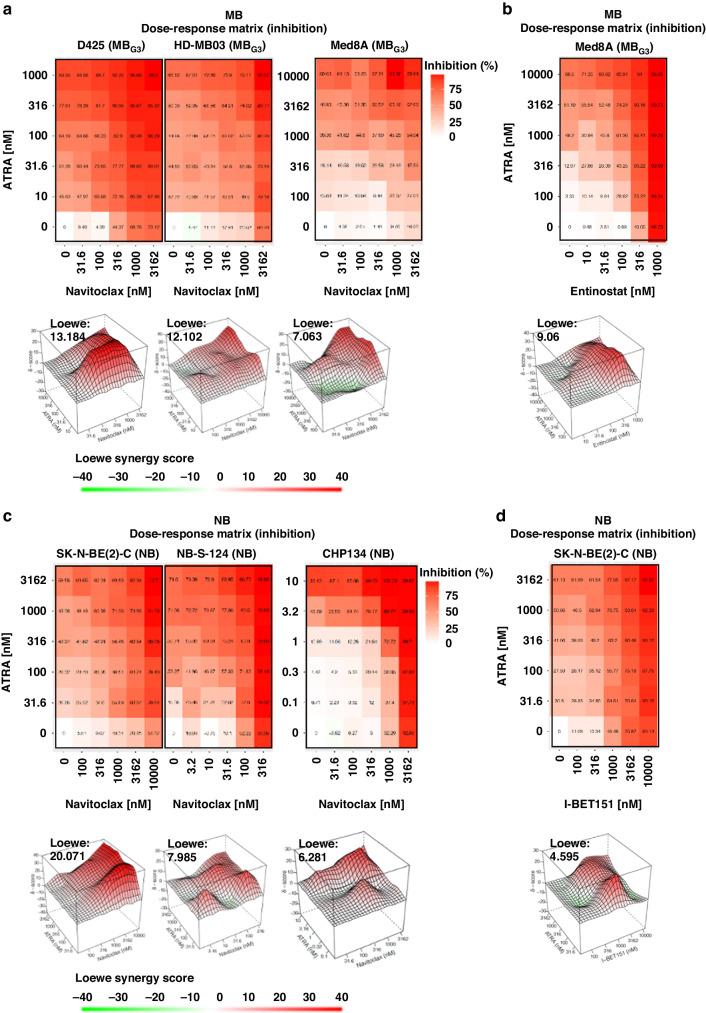
Retinoic acid/navitoclax cotreatment synergistically inhibits viability in culture models of both MB_G3_ and NB. **a** %Inhibition heatmaps of 5 × 5 ATRA/navitoclax dose-response matrices and 3D synergy maps annotated with overall Loewe synergy scores in *N* = 3 MB_G3_ tumouroid cell cultures after 144 h of treatment. Red depicts positive and green negative Loewe synergy scores. Values larger than 10 are interpreted as synergistic, −10 to 10 as additive, and below −10 as antagonistic. **b** 5 x 5 ATRA/entinostat dose-response matrix and 3D synergy map of Med8A. **c** 5 x 5 ATRA/navitoclax dose-response matrix and 3D synergy maps in *N* = 3 NB tumouroid cell cultures. **d** 5 × 5 ATRA/I-BET151 dose-response matrix and 3D synergy map of SK-N-BE(2)-C.

Due to its experimental setup with the use of one single RA concentration, the results of this drug combination screen with the most promising compounds were confirmed in a setup suitable for synergism analysis. We analysed drug combinations through a 5 × 5 matrix design and a self-generated seven-ray design based on the respective IC50 concentrations of the combination partners for antiproliferative synergy in the 6 *MYC(N)-*amplified MB and NB cell lines. As top hits for RA-responsive models, we chose navitoclax, which is currently under investigation in clinical trials (e.g., NCT05192889 in a paediatric cohort), entinostat (MB specific) and I-BET151 (NB specific). A Loewe synergy score >10 indicated synergy for ATRA/navitoclax (Fig. 3a) and additivity for ATRA/entinostat (Fig. 3b) in MBs. For NBs, additivity to synergy was indicated for ATRA/navitoclax (Fig. 3c), and additivity was indicated for ATRA/I-BET151 (Fig. 3d). The highest values were obtained for the ATRA/navitoclax combination in the MB_G3_ models D425 (13.18) and HD-MB03 (12.10) and the NB_MYCN_ model SK-N-BE(2)-C (20.07). The ray design analysis confirmed these results (Supplementary Fig. S9).

### RA-induced gene expression patterns in *MYC(N)*-amplified MB and NB involve differentiation and regulation of proliferation and apoptosis

**Fig. 4:**
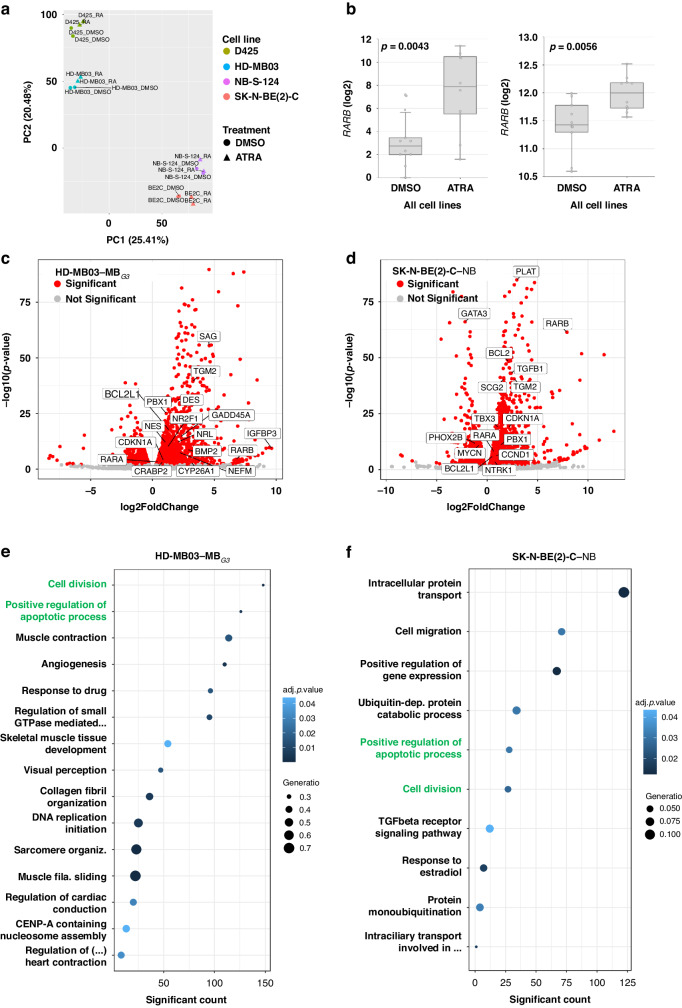
Retinoic acid induces transcriptomic changes in apoptotic regulation and differentiation. **a** Principal component analysis of expression patterns from RNA sequencing of *N* = 2 MB_G3_ and *N* = 2 NB culture models treated with ATRA (MB 0.5 µM/ NB 1 µM) or DMSO solvent for 144 h. Two replicates were performed for each condition. **b** Boxplots of *RARB*/*RARA* expression (log2) in all five cell lines comparing DMSO with 144 h ATRA treatment. **c**, **d** Volcano plots of differentially expressed genes upon 144 h of ATRA treatment depicting log2-(fold change) and -log10(p-value) in HD-MB03 and SK-N-BE(2)-C cells, respectively. Significantly up- or downregulated genes are depicted in red, not significant in grey. Selected genes of interest are annotated. **e**, **f** Significantly enriched GO terms upon 144 h of ATRA treatment in HD-MB03 and SK-N-BE(2)-C cells, respectively. GO terms are ordered by count of significantly differentially expressed genes, dot size represents gene ratio (proportion of significant genes in the respective GO term), and colour indicates the adjusted *p*-values. Overlapping GO terms are highlighted green.

To assess the impact of RA treatment on RA-responsive tumour cells on a transcriptional level, two *MYC*-amplified MB_G3_ and two *MYCN*-amplified NB models were chosen for bulk RNA sequencing. Cells were sequenced after 144 h of treatment with ATRA (1 µM in NB, 0.5 µM in MB) or solvent control. While the principal component analysis (PCA) dimension reduction indicated that the largest expression differences depend on the entity, followed by the cell line and last, the treatment condition (Fig. 4a), there are also shared response patterns to RA treatment. On a single gene level, RA induced the expression of genes involved in RA signalling (e.g., *RARB* and *RARA*, Fig. 4b) and metabolism, cell cycle regulation, and apoptotic regulation. In both NB and MB cell lines, the induction of genes involved in differentiation was observed. While RA induced signs of neuronal differentiation in NB, biochemical markers of both neuronal and myogenic differentiation were observed in MB cell lines upon RA treatment (Fig. 4c, d).

To identify the biological processes underlying the transcriptional changes, we performed gene ontology (GO) analysis, which revealed significant deregulation of the biological processes cell division, gene expression, positive regulation of apoptotic process, and drug response in both MB and NB models. In line with the induction of differentiation marker genes, MB cell lines were also found to be enriched in GO terms linked to myogenic differentiation (Fig. 4e, f). Overall, RA treatment led to gene expression changes implying hampered cell division, reduced proliferation, signs of differentiation and positive regulation of apoptotic processes.

### Combining RA treatment with BCL-X_L_ inhibition leads to a shift from differentiating effects to increased apoptosis

**Fig. 5:**
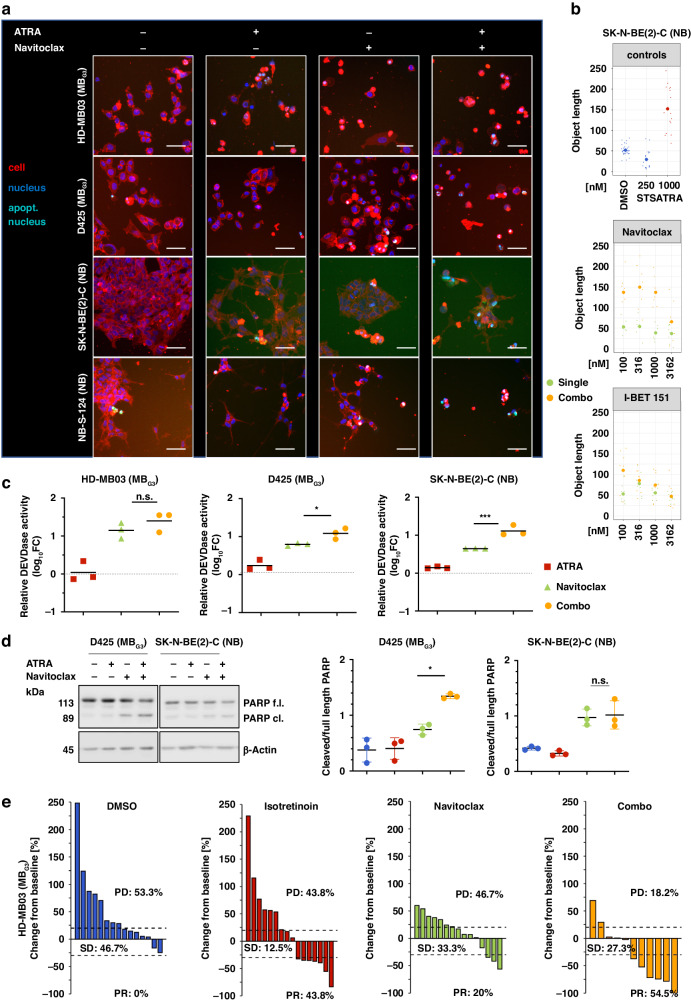
ATRA differentiating effects shift to increased induction of apoptosis in combination with navitoclax. **a** Representative images from high-content fluorescence microscopy of HD-MB03, D425, SK-N-BE(2)-C and NB-S-124 after 72 h of treatment with DMSO control, 500/1000 nM ATRA (MB/NB), 1000/3162 nM navitoclax (MB/NB), and a combination of both. Blue fluorescence shows nuclei, red shows cell bodies, and green (in overlay with blue: turquoise) shows apoptotic nuclei. Scale bar: 100 µm. **b** Graphs depicting the median object length of the cell including the cellular neurite-like protrusions after 72 h of DMSO, death control STS (staurosporine), ATRA, navitoclax, and I-BET151 treatment in SK-N-BE(2)-C cells. Green indicates single treatment, yellow indicates combination treatment. *N* = 3. **c** Log10-fold change of relative DEVDase activity compared to DMSO control in HD-MB03, D425, SK-N-BE(2)-C. Cells were treated with 500/1000 nM ATRA (MB/NB, red), 1000/3000 nM navitoclax (green), or combination (yellow) for 48 h. The bar indicates the corresponding mean. *N* = 3. **d** One representative Western blot of SK-N-BE(2)-C and D425 cells treated with 500/1000 nM ATRA (D425/SK-N-BE(2)-C), 1000/3000 nM navitoclax (green), or combination (yellow) for 72 h. PARP protein can be seen in its full-length (f.l.) and cleaved (cl.) version. Actin was used as a loading control. Dot plots on the right depict the ratio of cleaved to full-length PARP. The bar indicates the mean. *N* = 3. **e** Waterfall plots representing the change from baseline growth of individual HD-MB03 zebrafish embryo medulloblastoma xenografts treated with DMSO (blue), 500 nM isotretinoin (red), 10 µM navitoclax (green), and combination (yellow) for 48 h. Each bar represents a single xenograft tumour. DMSO, *N* = 15; isotretinoin, *N* = 16; navitoclax, *N* = 15; combo, *N* = 11. PD progressive disease, SD stable disease, PR partial response.

To visualise and quantify changes in cell morphology and cell death of two MB_G3_ (D425, HD-MB03) and NB_MYCN_ (SK-N-BE(2)-C, NB-S-124) models, high-content confocal fluorescence microscopy was used with fluorescent dyes for cell membrane (CellMask, red), nuclei (Hoechst, blue) and nuclei of apoptotic (caspase-3/7 active) cells (green). In line with previous findings of the effect of retinoids on NB morphology [21], a single RA treatment induced the formation of larger cell bodies and neurite-like cellular protrusions, a morphologic sign of differentiation. Longterm morphology assays confirmed these morphological changes for MB and NB cells, but not HGG nor EPN cells (Fig. 5a, Supplementary Figs. 10–13). In combination with navitoclax, the cellular morphology shifted from this differentiated phenotype to programmed cell death, as evidenced by decreased formation of neurite-like protrusions (Fig. 5b, Supplementary Figs. 10–11) and an increase in the fraction of apoptotic cells and fragmented nuclei (Supplementary Figs. S13, S14E, S15A) compared to RA single treatment. Moreover, the combinational treatment resulted in significantly decreased colony formation ability (Supplementary Fig. S15B). The fluorometric quantification of caspase-3/7 activity in the abovementioned *MYC(N)-*driven models, confirmed that treatment with navitoclax increased the number of apoptotic cells, which was enhanced when combined with RA in cell lines of both entities (Fig. 5c, Supplementary Fig. S14E). Consistently, navitoclax alone and especially RA/navitoclax cotreatment resulted in substantial apoptotic PARP cleavage (Fig. 5d). To further characterise the combination treatment with organoid-like patient-derived models, another round of high-content imaging was performed. The models (one *MYC*_amp_-MB_G3_, one NB, one EPN) were seeded as three-dimensional tumouroids. Treatment started three days after seeding and consisted of two cycles of four days presence followed by three days absence of drug for 14 days in total. The combination treatment substantially decreased spheroid sizes in MB_G3_ and NB models, but not in the EPN model (Supplementary Fig. S16). Notably, a RA non-responsive *MYC*_amp_-MB_G3_ organoid model did not reveal resistance-breaking combination benefits with navitoclax in the metabolic activity assay, supporting the conclusions that only RA responsive cells profit from RA/navitoclax cotreatment and that *MYC(N)* amplification alone does not predict RA sensitivity reliably (Supplementary Fig. S17A).

The combination treatment substantially impaired tumour growth in vivo. We transplanted either fluorescently labelled HD-MB03 or NB-S-124 cells into the yolk sac of zebrafish embryos and started treatment 24 h after injection for 48 h with solvent control, RA, navitoclax and combination. We applied the RECIST (Response Evaluation Criteria in Solid Tumours) criteria adapted to zebrafish for the definition of partial response (PR, tumour size ≥ 30% below basal value) and progressive disease (PD, tumour size ≥ 20% above basal value) [48]. While most tumours grew progressively in the control group (53.3% PD MB, 44.0% PD NB), less growth occurred in the single treatments with retinoic acid (43.8% PD MB, 21.4% PD NB) and navitoclax (46.7% PD MB, 32.1% PD NB). For the MB_G3_ model with the combination treatment more than half of the tumours decreased significantly in size (54.5% PR, partial response) and the number of tumours with progressive growth decreased significantly (18.2% PD). Combination treatment, thus, successfully led to a disease control rate (DCR: SD + PR) of over 80% of cases and achieved tumour regression (PR) in over 50% of cases (Fig. 5e). Similar results were obtained for the NB-S-124 NB model, where the combination treatment led to a DCR of 92% (Supplementary Fig. S17B).

### Medulloblastomas - in contrast to neuroblastomas - mainly depend on BCL-X_L_

**Fig. 6:**
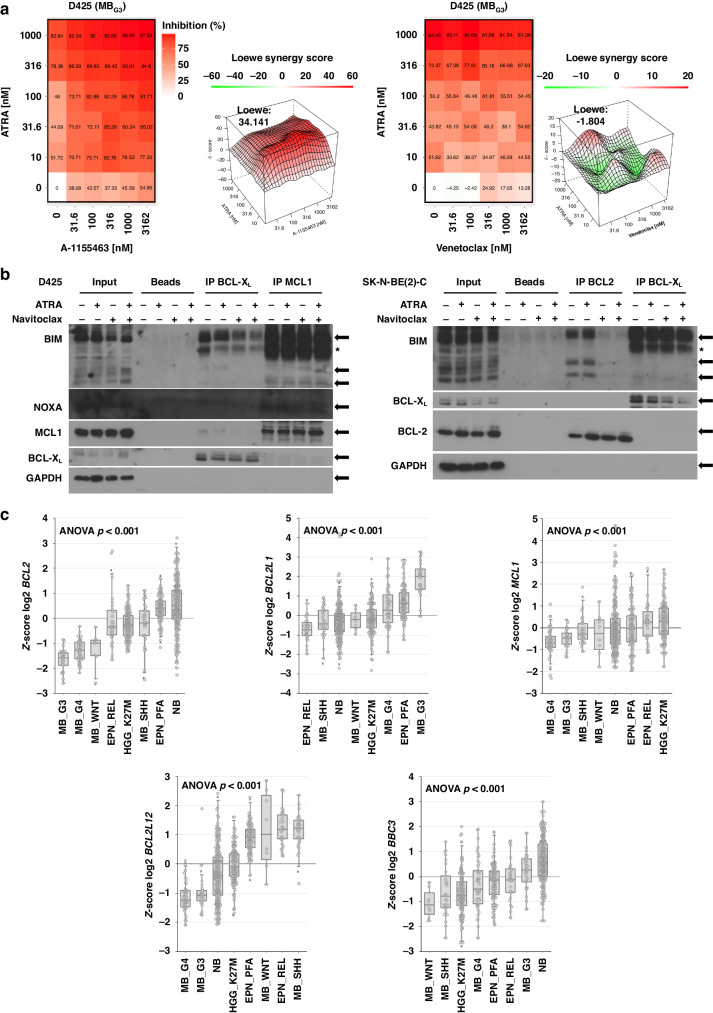
BCL-X_L_ is an important targetable vulnerability in MB_G3_. **a** %Inhibition heatmaps of 5 × 5 ATRA/A-1155463 (selective BCL-X_L_ inhibitor) and ATRA/venetoclax (selective BCL-2 inhibitor) dose-response matrices and 3D synergy maps annotated with overall Loewe synergy scores of D425 cells after 144 h of treatment. Red depicts positive and green negative Loewe synergy scores. Values larger than 10 are interpreted as synergistic, −10 to 10 as additive, and below −10 as antagonistic. **b** Representative immunoprecipitation plots of BCL-X_L_ and MCL-1 in D425 and BCL-2 and BCL-X_L_ in SK-N-BE(2)-C cells and detection of selected protein interaction partners. Cells were treated for 48 h with DMSO, 500/1000 nM ATRA (MB_G3_/NB), 1000/3000 nM navitoclax, and a combination thereof. GAPDH served as loading control in the input lysate. *N* = 3. **c** Boxplots of log2 z-score expression of anti-apoptotic genes *BCL2*, *BCL2L1*, and *MCL1* and putative BH3 mimetic response biomarkers *BCL2L12*, and *BBC3* as measured by RNA sequencing from *N* = 2288 relapsed paediatric tumours from the INFORM study. Boxplots depict selected entities of paediatric nervous system tumours. EPN_PFA posterior fossa ependymoma A, EPN-REL ZFTA-fusion positive ependymoma, HGG_K27M K27M-mutated high-grade glioma, MB_G3 Group 3 medulloblastoma, MB_G4 Group 4 medulloblastoma, MB_SHH sonic hedgehog medulloblastoma, MB_WNT wingless medulloblastoma, NB neuroblastoma.

To further narrow down the role of the navitoclax target proteins BCL-2 and BCL-X_L_ in combination with RA treatment, we performed a synergy analysis of the selective BCL-2 inhibitor venetoclax and selective BCL-X_L_ inhibitor A-1155463 combined with RA in MB_G3_ D425 cells. Here, venetoclax demonstrated neither single-agent efficacy nor synergy in the RA combination (Loewe synergy score −1.80). A-1155463, on the other hand, was both active and highly synergistic with RA (Loewe synergy score 34.14) (Fig. 6a). Thus, the combined benefit of RA/navitoclax in MB_G3_ is likely to be caused by targeting BCL-X_L_ and not BCL-2.

To assess interactions between pro- and anti-apoptotic proteins in single- and cotreatment conditions, immunoprecipitation (IP) was performed in D425 (MB_G3_) and SK-N-BE(2)-C (NB_MYCN_) cells after 48 h of treatment (Fig. 6b). As D425 cells depend on BCL-X_L_ and hardly express BCL-2, IPs were performed for the anti-apoptotic proteins BCL-X_L_ and MCL-1. The pro-apoptotic BIM, which is mainly expressed in the BIM-EL form and less as BIM-L/S in D425, was associated with BCL-X_L_. Upon treatment with navitoclax, some displacement of BIM from BCL-X_L_ was observed. In SK-N-BE(2)-C cells, which strongly express BCL-2, IPs were performed for BCL-2 and BCL-X_L_. Here, BIM was mainly bound to BCL-2 and hardly any to BCL-X_L_. Treatment with navitoclax displaced BIM-L and BIM-S from BCL-2. Thus, the on-target activity of navitoclax is evident in both MB and NB models, leading to substantial apoptosis when combined with RA treatment.

To explore the role of BCL-2, BCL-X_L_ and MCL-1 in a large paediatric patient pancancer cohort, we investigated their expression in RNA sequencing of *n* = 2288 patient samples from the INFORM registry. The international precision oncology programme INFORM enrols relapsed/refractory paediatric cancer patients for comprehensive molecular analysis. It revealed that NB was the solid tumour with the highest expression of *BCL2*, while MB_G3_ demonstrated among the lowest *BCL2* expression. *BCL2L1* (BCL-X_L_), on the other hand, was most highly expressed by MB_G3_, while NB tumours showed average expression. The below average expression of *MCL1* suggests that this anti-apoptotic protein may play only a minor role in relapse of the embryonal tumours MB_G3_ and NB (Fig. 6c). As mere expression of the anti-apoptotic target proteins has not been clearly demonstrated to predict response to BH3-mimetic treatment, we investigated the expression of the recently suggested predictive biomarkers *BCL2L12* and *BBC3* for paediatric tumours [31]. Consistently, MB_G3_ and NB tumours of the INFORM cohort expressed *BCL2L12* at comparatively low levels and *BBC3* at above-average levels (Fig. 6c; Supplementary Fig. S18). Comparing the four canonical MB subgroups in detail, Group 3 tumours both expressed *BCL2L1* (BCL-X_L_) and *BBC3* at significantly higher levels than the other subgroups and showed significantly lower *BCL2L12* expression than the MB_SHH_ and MB_WNT_ subgroups (Fig. 6c).

Overall, these results suggest that RA and navitoclax cotreatment not only elicits strong antitumoural effects in the MB_G3_ and NB preclinical models studied here, but also may provide a novel treatment option for relapsed patients with these high-risk cancers.

## Discussion

MB_G3_ and NB are embryonal tumours that result from impaired differentiation of neuronal lineage progenitors during fetal development of the cerebellum and sympathetic nervous system, respectively [12, 49]. Despite highly intensive treatment approaches, the current standard of care for these diseases does not yield acceptable outcomes in high-risk and relapsed cases [7, 13]. Therefore, we investigated the potential of the differentiation-inducing drug RA and sought to identify a combinatorial compound that might efficiently enhance its effects.

In accordance with other preclinical investigations we demonstrated RA sensitivity in NB and MB_G3_, while MB_SHH_ models show a very limited response [20, 22, 50, 51]. Conversely, paediatric HGGs and PFA- and ZFTA fusion-positive EPN models resisted antitumoural RA effects, contrary to one previous study of two PFA cultures [24]. This might be explained by the three-dimensional tumouroid model used in our study, which mimics drug-resistant phenotypes similar to the in vivo situation. Our data indicate that RA is especially effective in tumour subtypes capable of neuronal differentiation and that RA treatment supports neuronal differentiation in MB_G3_ and NB as demonstrated by translational regulation of marker genes, and morphological changes in the neuroblastoma models, independent of the growth rate. In the transcriptomic data of NB cells, we observed changes consistent with the recently described RA-induced reprogramming of the adrenergic core regulatory circuit [52]. Our results also point toward RA-induced upregulation of anti-apoptotic members of the BCL-2 family, such as *BCL2* and *BCL2L1*, which are druggable with BH3 mimetics. Thus, RA treatment also induces novel targetable vulnerabilities, such as the BCL-2 family as evidenced by cotreatment with the BCL-2/BCL-X_L_ inhibitor navitoclax, which shifted the differentiated phenotype to cell death. RA/navitoclax effectively inhibited tumour growth in an in vivo zebrafish embryo xenograft model. This model is characterised by high evolutionary conservation of human genetic pathways, an immature immune system preventing rejection, and transparency allowing live visualisation of in vivo growth and migration [53]. The low amount of cells used per injection and cost- and time-effectiveness make it an attractive model for high-throughput precision oncology approaches [43, 48].

*BCL2L1* and markers of the BH3-mimetic response are highly expressed in relapsed MB_G3_ patients, suggesting clinical potential for BCL-X_L_ inhibition in these patients. Targeting the BCL-2 protein family provides a promising area of investigation for the treatment of paediatric nervous system tumours [27], as the anti-apoptotic BCL-2 family members BCL-2, BCL-X_L_, and MCL-1 are frequently upregulated in cancer cells, including MB and NB [26]. Of note, across various cancers, *MYC* amplification is often associated with upregulation of BCL-X_L_ and MCL-1, rescuing cells from *MYC*-induced apoptosis [26]. In NB, BCL-2 plays an important role in the RA response, with low BCL-2 expressing cells undergoing RA-mediated apoptosis, while differentiating cells increase BCL-2 expression and consequently evade cell death [54]. RA-induced differentiation in NB has also been linked to decreased chemosensitivity to antineoplastic agents, such as topotecan, doxorubicin and cisplatin, which is mediated through BCL-2 and BCL-X_L_ upregulation [55].

BH3 mimetics have been investigated in many preclinical studies regarding their efficacy in paediatric nervous system tumours as single and combinatorial agents [27]. The combination of the investigational synthetic retinoid fenretinide with selective BCL-2 inhibition was shown to synergistically induce cell death and increase survival in an in vivo model through NOXA upregulation in NB cells that express high levels of BCL-2 [56]. The sensitivity of NB cells to selective BH3 mimetics targeting BCL-2, BCL-X_L_, and MCL-1, however, is heterogeneous and all three antiapoptotic proteins are equally relevant targets for apoptotic modulation [42]. Notably, selective BCL-X_L_ inhibition was most effective against primary patient-derived NB cells [42]. In addition, in a study of selective BH3 mimetics across 18 paediatric solid tumour cell lines, the selective BCL-2 inhibitor venetoclax was the least efficacious, and concomitant antagonism of BCL-X_L_ and MCL-1 produced the most pronounced induction of cell death [57]. In MB, BCL-X_L_ is a direct transcriptional target of the MB_G3_ master transcription factor *NRL* and has been described as a potential subgroup-specific vulnerability [58]. Indeed, targeting BCL-X_L_ can induce apoptosis in MB_G3_ cell culture and impede growth in orthotopic xenografts [58]. Furthermore, subsequent studies of BH3 mimetics in MB_G3_ demonstrated that navitoclax and selective inhibition of BCL-X_L_, but not BCL-2, while having modest single-agent antitumoural activity, render cells more vulnerable to the effects of cytotoxic stimuli, chemotherapy, and targeted therapeutics by increasing the induction of intrinsic apoptosis [59–61]. Consistent with these findings, our study provides evidence that effective targeting of apoptotic regulation in MB_G3_ is primarily achieved by antagonising BCL-X_L_ and should be combined with other treatment approaches, such as RA, that synergise with BH3 mimetics in inducing cell death. In NB, RA treatment may benefit most from combination with a BCL-2-/BCL-X_L_-inhibitor such as navitoclax or APG-1252, as both proteins seem to be involved in the RA response and provide relevant targets.

To evade apoptotic effects of oncogenic *MYC(N)*, tumour cells may upregulate pro-survival BCL-2 family members or suppress *TP53* signalling. In colorectal cancer cells it has been shown that p53, which undergoes entinostat induced post-transcriptional modifications, can directly interact with BCL-2-family proteins to trigger apoptosis [62]. In this study, we did not investigate the treatment-induced protein interactions of p53, however, *TP53* status did not seem to correlate with distinct response patterns to the investigated apoptotic induction.

Besides BH3 mimetics we identified therapeutic potential in combining the HDAC inhibitor entinostat with RA in MB_G3_ models. Noteworthy, the class I inhibitor entinostat was far more beneficial for the combination with ATRA as the pan-HDAC inhibitor vorinostat, but no clear class-specific combination effect was shown. Furthermore, class I HDAC inhibition can also result in adverse combination effects with RA through antagonism between differentiation and cytotoxicity in other entities [63–66]. In acute promyelocytic leukaemia cells, RA-induced BCL-X_L_ activity has been described as cytoprotective against HDAC class I inhibition but less so against pan-HDAC inhibition [63]. Notably, a recent MB_G3_ cell culture study demonstrated synergistic activity between BCL-X_L_-inhibition and entinostat [61]. Thus, further studies would be needed to provide clear evidence and predictive biomarkers for clinical translation in selected cancer (sub-)entities.

From a clinical perspective, it is important to note that the RA/navitoclax cotreatment concentrations that synergistically decreased MB and NB viability to relevant levels (<50%) are achievable in patients [18, 67]. While there are already decades of experience with isotretinoin/13-*cis*-RA therapy and its safety and efficacy in high-risk NB patients [15, 18], its use has been limited to experimental approaches in MB. However, recently, Leary et al. demonstrated that oral isotretinoin treatment analogous to NB protocols could be safely incorporated into an intensive cytotoxic chemotherapy regimen for infant embryonal brain tumours, including MB_G3_ alongside the HDAC inhibitor vorinostat, with acceptable toxicity [68]. Moreover, a randomised-controlled Children’s Oncology Group trial tested carboplatin and isotretinoin in high-risk MB and supratentorial primitive neuroectodermal tumours [69]. However, the isotretinoin treatment arm was closed before molecular subgroup analyses were introduced into diagnostic routine and no subgroup-specific analyses were reported [69]. It is conceivable that RA might only provide benefit for a certain subgroup of MB patients, which according to the evidence provided here would most likely be found within MB_G3_. In such a group, RA would likely exert its strongest potential when given with a suitable targeted therapeutic, rather than conventional chemotherapy.

Navitoclax is known to induce dose-limiting thrombocytopenia, which has been attributed to its targeting of BCL-X_L_ [67], yet intermittend or lower navitoclax dosing [70] and novel dual BCL-2-/BCL-X_L_ [71] inhibitors show promising clinical potential, as these strategies may provide better tolerance. Nevertheless, navitoclax was chosen for our investigations as it is the only BCL-2-/BCL-X_L_-inhibitor with described pharmocokinetics in the paediatric oncology population [70], where it is also currently investigated in ongoing clinical trials (e.g., NCT05192889 or NCT05740449). Finally, the penetration of BH3 mimetics through the BBB into the CNS is still in question. A disruption of the BBB, as is observed in paediatric brain tumours clinically, however, might provide a way for BH3 mimetics to treat these cancer cells despite high molecular weights [61, 72].

Unlike with current first line therapies in paediatric oncology, there is an unmet clinical need for innovative treatment strategies in recurring childhood cancer, which shows poor survivaI [3, 29, 31, 45]. Thus, many of the culture models in our study are derived from relapsed disease, which is often characterised by accumulating genetic mutations confering treatment resistance e.g. through p-glykoprotein activity [41] and changed BCL-2 family dependencies [73]. As translation of newly identified treatment approaches to early phase clinical trials in paediatric oncology mainly focuses on treating recurrent or refractory disease, we investigated potential predictive biomarkers in the comprehensive INFORM cohort. Nevertheless, our study does not allow for a clear conclusion regarding treatment potential in primary as compared to relapsed disease, as primary in contrast to relapsed tumours are characterised mainly by amplifications and deletions.

In conclusion, we demonstrate that RA treatment modulates apoptotic regulation in MB_G3_ cells and subsequently increases vulnerability to selective BCL-X_L_ inhibition. While both compounds show limited single-agent activity, RA/BCL-X_L_-inhibition cotreatment synergistically induces apoptotic cell death and improves in vivo antitumoural efficacy. Together with molecular data from relapsed patients, our results suggest that BCL-X_L_ is a particularly important target in MB_G3,_ and the combination treatment investigated here may provide clinical benefit in these patients.

### Supplementary information

Supplemental Material

## Data Availability

Gene expression data are available through the web-based gene expression analysis R2 (R2: microarray analysis and visualisation platform; http://r2.amc.nl) platform: Medulloblastoma / Neuroblastoma RA - Oehme - 20 - rsem - gencode19; 2021-12-01.
