# Supplementary material for: Synergy of retinoic acid and BH3 mimetics in MYC(N)-driven embryonal nervous system tumours
Source: Br J Cancer. 2024 Jun 28;131(4):763–77. doi: 10.1038/s41416-024-02740-5 (PMC11333474; doi:10.1038/s41416-024-02740-5)
Supplement: Supplementary file 1 — Supplemental Material [file 41416_2024_2740_MOESM1_ESM.pdf]

Synergy of Retinoic Acid and BH3 Mimetics in *MYC(N)*-Driven Embryonal Nervous System Tumors

Supplementary Materials and Methods

Cell culture

The following human neuroblastoma cell lines were applied: SK-N-BE(2)-C (RRID: CVCL_0529, European Collection of Authenticated Cell Cultures, ECACC, Salisbury, UK) and SH-SY5Y (RRID: CVCL_0019, German Collection of Microorganisms and Cell Cultures, DSMZ, Braunschweig, Germany) were maintained using Dulbecco's modified Eagle's medium (DMEM with L-glutamine and 4.5 g/L glucose; Gibco, Thermo Fisher Scientific Inc., Walthaem, MA, USA) supplemented with 10% heat-inactivated fetal calf serum (FCS; Sigma-Aldrich, St. Louis, MO, USA) and 1% nonessential amino acids 100X (NEAA; Lonza, Basel, Switzerland). CHP134 cells (RRID: CVCL_1124, kindly provided by Frank Westermann, DKFZ, Heidelberg, Germany) were cultured in Roswell Park Memorial Institute medium 1640 (RPMI; Gibco) supplemented with 10% FCS and 1% NEAA. Neuroblastoma short-term culture NB-S-124 (kindly provided by Frank Westermann) were cultured as tumor spheres in serum-free Neurosphere-Medium (NSM) consisting of Neurobasal A medium (Gibco) with 2% B-27 Supplement Minus Vitamin A (Gibco), 20 ng/ml human bFGF (PeproTech Inc., Rocky Hill, NJ, USA), 20 ng/ml human EGF (PeproTech Inc.), 2 mM L-glutamine (Gibco) and 2 µg/ml Heparin (Sigma-Aldrich).

Four human medulloblastoma cell lines were used: HD-MB03 (RRID: CVCL_S506[1]) was cultured in RPMI 1640 medium, and D425 (RRID: CVCL_1275, American Type Culture Collection, ATCC, Manassas, VA, USA) was cultured in Improved Minimum Essential Medium (IMEM Zinc option; Gibco), each with 10% FCS and 1% NEAA. Med8A (RRID: CVCL_M137, kindly provided by R. Gilbertson, Memphis, TN) and ONS-76 (RRID: CVCL_1624, Institute of Fermentation, Osaka, Japan) were maintained using DMEM with 10% FCS and 1% NEAA.

As pediatric ependymoma models, DKFZ-EP1NS [2] were cultured in NSM and Epd210FH (kindly provided by James Olson, Brain Tumor Resource Lab, Seattle, WA, USA) in NeuroCult NS-A Basal Medium (Human) with NS-A Proliferation Supplement (STEMCELL Technologies, Vancouver, Canada) supplemented with 75 ng/ml bovine serum albumin (BSA, Invitrogen, Thermo Fisher Scientific Inc.), 1% penicillin/streptomycin (Sigma-Aldrich), 2 mM L-glutamine, 20 ng/ml human bFGF and 20 ng/ml human EGF.

The following human pediatric high-grade glioma models were used: KNS-42 (RRID: CVCL_0378) and SJ-GBM2 (RRID: CVCL_M141) (both kindly provided by David T.W. Jones, DKFZ) cell lines were cultured using DMEM with 10% FCS and 1% NEAA. Patient-derived models SU-DIPG-13 (RRID: CVCL_IT41) and SU-DIPG-25 (RRID:CVCL_C1N0) (both kindly provided by Michelle Monje, Stanford, CA, USA) were cultured as neurospheres in serum-free tumor stem medium (TSM) containing 1:1 neurobasal A medium and DMEM/F-12 (Gibco) supplemented with 1% HEPES buffer solution (1 M) (Gibco), 1% sodium pyruvate MEM (100 mM) (Gibco), 1% NEAA, 2 mM L-glutamine, 1% penicillin/streptomycin, 2% B-27 supplement

minus vitamin A, 20 ng/ml bFGF, 20 ng/ml human EGF, 10 ng/ml human PDGF-AA (PeproTech Inc.) and 2 µg/ml heparin. Long-term cultures (LTC) were established from primary tumor samples from the INFORM program as previously described and cultured as free-floating and semiadherent spheroids using [3-5]. The following INFORM-derived *ex vivo* cultures were used: INF_R_1073_relapse1_LTC (HGG, wild-type) and INF_R_859_primary_LTC (= DKFZ-BT278, HGG, H3_G34R). Standard culture conditions of a humidified atmosphere at 37°C and 5% CO_2 were applied. All cultures were confirmed to be negative for mycoplasma, viruses and cross-contamination by routine multiplex cell contamination tests (McCT, Multiplexion, Heidelberg, Germany) and DNA fingerprinting authentication (DSMZ).

PDX-derived MB_{G3} fresh tissue spheroid culture: Dissociation of the *MYC*-amp MB_{G3} Med_2112fh PDX tumor was performed essentially as described in Peterziel *et al.*[6] (protocol for brain tumors and brain metastases). As the tissue readily dissociated upon mechanical mincing, enzymatic dissociation was omitted. The cell suspension was filtered through a 100 µm cell strainer (431752; Corning) prior to counting and determination of cell viability, and cultured as free-floating spheroids in NeuroCult complete (NeuroCult NS-A Proliferation Kit (Human); 90% basal medium + 10% supplement (05751; StemCell Technologies, Vancouver, Canada), H-EGF (f.c. 20 ng/ml; AF-100-15B, Thermo Fisher Scientific, Waltham, MA USA), H-FGF-basic-154 (f.c. 20 ng/ml; Thermo Fisher Scientific, Waltham, MA USA).

PDX-derived MB_{G3} tumoroid culture: The *MYC*-amplified MB_{G3} patient-derived Orthotopic Xenograft (PDOX) RCMB28 tumor cells were cultured as 3D tumoroids in suspension plates using PDX medium, containing 50% Advanced DMEM (Gibco 12491015) and 50% Neurobasal (Gibco 21103049) supplemented with, N2 (Gibco 17502001), B27 (Gibco 12587001), HEPES buffer (Gibco 15630056), GlutaMAX (Gibco 35050061), Penicillin-Streptomycin (Gibco 15140122), Heparin Solution (Stemcell Technologies 07980), epidermal growth factor (EGF) 20 ng/ml (Peprotech AF-100-15) and fibroblast growth factor (FGF2) 10 ng/ml (Peprotech 100-18B). Tumoroids were cultured in a 37 °C, 5% CO2 incubator and medium was refreshed every 3-4 days and cells were split every 2 weeks.

Metabolic activity assays

Adherent cells were detached using trypsin (Gibco). Spheroid cultures were dissociated using TrypLE Express (Gibco) or Accutase (Sigma-Aldrich) for EPD210FH and Versene (Gibco) for NB-S-124. A Vi-CELL XR automated cell counter (Beckmann Coulter, Krefeld, Germany) was used for automated trypan blue staining to count viable cells. Cells were seeded as three-dimensional spheroids in U-shaped round-bottom 384-well ultralow attachment plates (Corning Inc., Corning, NY, USA). A TECAN D300e (TECAN, Männedorf, Switzerland) was used to dispense drugs onto plates after seeding. For treatment, wells were randomized using the randomization feature in the TECAN D300e Control software. For antitumoral dose-response activity of RA, a ½ Log distribution was used over a concentration range of 1 nM to 100 µM. Metabolic activity was assessed after 144 h by adding 15 µl of ATP-based CellTiter-Glo 2.0 Cell Viability Assay (Promega, Madison, WI, USA) per well and shaking the plates for 5 minutes at

400 rpm. After incubation for 10 minutes, PheraStar (BMG Labtech, Ortenberg, Germany) or TECAN Spark (TECAN) plate readers were used to measure bioluminescence.

Medium-throughput drug combination screening

Combination drug screens were performed) by simultaneous monotherapy and combination library screens. Concentration ranges of 76 clinically relevant drugs (30 kinase inhibitors, 22 conventional chemotherapeutics, 11 differentiating agents/epigenetic drugs, 8 apoptotic modulators, 3 rapalogs and 2 other) [6] were imprinted as duplicates onto sets of three round-bottom 384-well plates (ready-to-go assay plates) at the High Throughput Biomedicine Unit, Institute for Molecular Medicine Finland (FIMM, Helsinki Institute of Life Science, Helsinki, Finland). To screen for combination effects, a single fixed concentration of 1 µM/500 nM/10 nM RA (NB cultures/primary brain tumors/CHP-134) or solvent control was added to one of two sets of ready-to-use assay plates per cell line. Cells were seeded on wells with predispensed drugs and underwent incubation and readout as described above.

Mono- and combination therapy responses were analyzed by calculating the multiparametric modified drug sensitivity scores (DSS_{asym}) through asymmetric curve fitting with the iTReX (interactive Therapy Response eXploration, https://itrex.kitz-heidelberg.de) application [7]. To estimate potential combination benefits, the differential combination DSS ($dcDSS_{asym}$) was used, which was calculated by subtracting the monotherapy sensitivity (DSS_{asym} (mono)) from the combination sensitivity (DSS_{asym} (combo)). Drugs with a $DSS_{asym} > 10$ and a $dcDSS_{asym} > 2$ were identified as preliminary hits for potential effectiveness and synergism [7].

Caspase-3/7 activity assay

To measure caspase-3/7 activity, the Caspase-3/7 Fluorometric Assay (BioVision, Abcam, Cambridge, UK) was performed following the manufacturer's instructions. Cells were seeded on 10 cm dishes and, 24 h later, treated as indicated. After 48 h, cells were collected using trypsin, resuspended in cell lysis buffer, and stored overnight at -80°C. Protein content was assessed using the colorimetric Pierce BCA Protein Assay-Kit (Thermo Fisher Scientific Inc.). Samples were diluted to equal protein amounts, and 2x reaction buffer containing 10 mM dithiothreitol (DTT) and caspase-3 substrate DEVD-AFC were added. Samples were then transferred to black 96-well plates. Upon substrate cleavage, submitted fluorescence was recorded every 5 minutes for 3 h using a FLUOstar OPTIMA plate reader (BMG Labtech) at 37°C and excitation/emission filters at 380/520 nm. Signal slope/minute values were normalized to the DMSO control to determine relative caspase-3/7 activity.

Trypan blue assays

Cells were seeded on 6-well plates and treated as indicated, after which cells were harvested and pooled with the corresponding supernatant, centrifuged, resuspended in medium and finally underwent automated trypan blue staining using the Vi-Cell XR Cell Viabiliy Analyzer (Beckman Coulter, Krefeld, Germany). Stained (dead) and unstained (viable) cells were counted by the Vi-Cell XR 2.03 software.

Western blot analysis

Cells were seeded onto 10 cm dishes, incubated for 24 h and treated for 72 h as indicated. Cellular lysis with sodium dodecyl sulfate (SDS) buffer (Tris 0.5 M, hydrochloric acid (HCl) (pH 6.8), SDS 2%, 87%-glycerol 10%, dithiothreitol 1 mM) was followed by protein denaturation at 95°C. Samples of equal protein amounts then underwent electrophoretic separation by SDS-PAGE and protein transfer to polyvinylidene fluoride (PVDF) membranes by semidry electroblotting. After application of blocking solution (Tris-buffered saline, nonfat dry milk 20%, FCS 20%, bovine serum albumin 3%, normal goat serum 1%, Tween 20 0.2%), membranes were incubated with primary antibodies overnight at 4°C followed by peroxidase-conjugated secondary antibody. The following primary antibodies were used: anti-β-actin (4967, Cell Signaling, Danvers, MA, USA), anti-GAPDH (JC1682928, Millipore, Burlington, MA, USA), anti-BID (2002, Cell Signaling) and anti-PARP (poly(ADD)ribose polymerase) (9542, Cell Signaling).

High-content imaging of patient-derived tumoroids

For the imaging experiments, the cells were harvested and dissociated as described in Peterziel *et al.*[6], counted, and labeled by adding tetramethylrhodamine (TMRE¸ f.c. 100 nM; ab113852, abcam) to the cell suspension. Cells were seeded at a density of 500 cells and 60µl medium per well (TSM complete for INF_R_1632_relapse1_PDX_LTC [NB] and INF_R_1887_relapse1_LTC [EPN]; NeuroCult complete for Med_2112fh_PDX_FTC [MB_{G3}]) in 384 round bottom ultra-low attachment spheroid microplates (3830; Corning) to allow formation of spheroids. Treatment started three days after seeding and consisted of two cycles of four days presence of drugs (ON) followed by three days absence of drugs (OFF) for 14 days in total (ON-OFF-ON-OFF). For navitoclax the concentration range covered eleven half-logarithmic dilutions (10 µM – 0.1 nM), ATRA was dispensed at 500 nM (INF_R_1887_relapse1_LTC and Med_2112fh_PDX) and 1 µM (INF_R_1632_relapse1_PDX_LTC), respectively as single drug and in combination with the navitoclax concentration range. Wells with 0.1% DMSO were included as minimum effect controls. Staurosporine (1 µM and 250 nM) was added as positive control, wells with 100 µM BztCl served as maximum effect control. All drugs were dispensed with a D300e digital dispenser (Tecan, Männedorf, Switzerland) at day 0 and day 7). Treatment OFF (day 4 and day 11) was achieved by replacing 3/4 of the medium (45 µl) with fresh medium (without drugs) with an Integra ASSIST PLUS pipetting robot (INTEGRA Biosciences AG, Zizers, Switzerland).

Images were acquired with an ImageXpress Micro Confocal imaging system (Molecular Devices, San Jose, CA, USA) using a 10X Plan Apo objective (one field per well) for six time points: day 0 (starting point; three days after seeding, first treatment day), day 2, day 4, day 7, day 9, day 11 and day 14. The acquired imaging TIFF files were uploaded to CellProfiler for subsequent image analysis. The TMRE-based spheroid area was measured in pixels for each well. The percentage change in spheroid area from baseline was calculated for each well and imaging day, using the following formula: ((dayX / day0) x 100) - 100. The calculated area change was plotted for each drug treatment using GraphPad Prism v10.2.1.

Cell growth and Drug Treatment Viability Assays of PDX-derived MB_{G3} Tumoroid

RCMB28 MB_{G3} tumoroids were collected and 500 cells were resuspended in fresh medium and plated in a volume of 40 µl in 384-well plates (Corning CLS3830) using the Multidrop Combi system dispenser (ThermoFisher Scientific) and allowed to form spheres in 24h. Tumoroids were treated with Nativoclax (0.1 µM , 0.25 µM and 0.5 µM) and ATRA (0.5 µM) using the Tecan D3003 Digital Dispenser. Cells were treated with DMSO as negative control and with Staurosporine (10 µM) (BIO-CONNECT BV 62996-74-1) as positive control. After 144 h treatment metabolic activity was measured by CTG readout as described above. Results were normalized to DMSO negative controls (100% viability) and Staurosporine positive controls (0% viability).

Immunoprecipitation

Cells underwent treatment for 48 h before undergoing flash freezing with liquid nitrogen and transfer to the partner institution for analysis. The following antibodies were used: anti-BCL-2 (M088701-2, Dako, Hamburg, Germany), anti-BCL-X_L (2762S, Cell Signaling), anti-MCL-1 (ADI-AAP-240F, Enzo, Farmindale, NY), anti-BIM (3183S, Cell Signaling), anti-NOXA (ALX-804-408, Enzo), anti-β-actin (A5441, Sigma-Aldrich) and anti-GAPDH (5G4-6C5, BioTrend, Köln, Germany).

Zebrafish lines, toxicity assays, embryo xenotransplantation, and treatment

AB strain wild-type zebrafish embryos were used. Before xenotransplantation, drug toxicity was assessed by determining the maximum tolerated dose (MTD) for 13-cis-RA and navitoclax. Toxicity assay was performed as described previously [8]. Briefly, the effect of compounds for behavior, morphological changes and death was imaged at day one and day three of experiment. Xenotransplanted embryos bearing HD-MB03 or NB-S-124 tumor cells labeled with CellTracker CM-DiI (Thermo Fisher Scientific) were placed in E3 medium supplemented with 1% N-Phenylthiourea (PTU, Sigma-Aldrich, Munich, Germany) and treated with either drugs or a solvent control. Tumor growth was assessed using a Zeiss LSM 710 confocal microscope (Zeiss, Oberkochen, Germany) and ZEN software (Zeiss, Oberkochen, Germany) before the drug exposure and 48 hours after treatment.

Patient-derived xenograft development

Xenograft of tumor cell suspension was carried out in immune-compromised mice (Nod-scid IL2Rgammanull) immediately following processing, NSG mice were obtained from the laboratory breeding colony (German Cancer Research Center). All mice were maintained in accordance with the National Institutes of Health (NIH) Guide for the Care and Use of Laboratory Animals with approval from the Fred Hutchinson Cancer Research Center Institutional Animal Care and Use Committee (IR no. 1457). Anesthesia, surgery and xenotransplantation was performed as described previously.[9] The cell suspension (4 µl; 100,000 cells in NeuroCult + 10% Proliferation Supplement) was implanted into the intracranial space by inserting a 10 µl Rainin Pipette-Lite fitted with a 2–10 µl ART tips barrier non-filtered pipette tip (Thermo Scientific, 2139). Mice implanted with patient-derived tumor

cells were monitored regularly for signs of tumor formation. On observation of symptoms mice were euthanized and tumor presence was confirmed visually during tissue resection.

Gene expression analysis R2

Available gene expression data for the brain tumor culture models used were derived from the following Affymetrix microarray datasets available on the R2 Genomics Analysis and Visualization Platform (http://r2.amc.nl): Brain Tumor (2023-01-23) - Kool - 123 - MAS5.0 - u133p2, which contains samples of various brain tumor models under differing culture conditions. Samples for our data analysis were extracted, where gene expression profiles under standard culture conditions as described above were available for the models investigated in this study. In the case of INFORM LTCs, gene expression profiles were derived from Pediatric Inform - Pilot / Registry - 1057 - MAS5.0 - u133p2. Out of the N=12 brain tumor culture models two were excluded because RA IC50 values could not be calculated due to no dose-dependent response (ONS-76, SU-DIPG-13) and two were excluded due to missing expression data (SJ-GBM-2, KNS-42). Neuroblastoma gene expression is derived from the R2 RNA sequencing dataset Neuroblastoma - Westermann - 39 - TMM - ensh37e75. As no SK-N-BE(2)-C expression was available in this dataset, the parental cell line SK-N-BE(2) was chosen as a surrogate.

R script Ray design

```
library(drc)

fit                 <-function(xt,y,x){                                                     # Function to fit a
loglogistic model and generate a prediction at values "x"
comfit              <-drm(y~xt, fct = LL.4(), lowerl = c(-100,0,0,0))
par                 <-summary(comfit)$coefficients[,1]
parall              <-summary(comfit)$coefficients
ydach               <-par[2]+(par[3]-par[2])/(1+exp(par[1]*log(x)-par[1]*log(par[4])))
return(list(comfit,par,ydach,parall))}

inversefunk<-function(theta,y){
erg                 <-theta[4]        *((theta[2]-y)/(y-theta[1]))^(1/theta[3])          # inverse of the log-logistic function
return(erg)}

#################### Read and prepare data set
##################################################################################################

setwd                                 ('<your path here>')
dat                 <-read.csv2       ("<your filename here>.csv",header=TRUE, sep = ";",dec = ",")

dat$ProportionA<-dat$ConcA/(dat$ConcA+dat$ConcB)
dat$ConcA[dat$ConcA==0]<-0.0001                                                      # Replace zeros with small
values to allow log scale
dat$ConcB[dat$ConcB==0]<-0.0001
dat$ConcA           <-log10(dat$ConcA)                                             # Take logs
dat$ConcB           <-log10(dat$ConcB)

faktor              <-unique(round(dat$ProportionA,6))[-1]                         # Proportions of Substance A in mixture
(absolute), remove DMSO
```

```
rays                <-unique(dat$Ray)[c(2,3,4,5,6,7,8,1)]                       # Rays used, first ray is no real ray but
DMSO control
k                   <-length(rays)-1                                            # Number of rays, DMSO is no ray on its
own

################### Fit loglogistic functions and generate predictions for all rays
##################################################################

dens                <-400                                                                  # Grid density for plot of
predicted dose response curve
x                   <-10^(c(-(4*dens):(10*dens))/dens)                          # Range of dose levels for prediction
xl                  <-length(x)


xall                <-list()                                                    # Summary vector to collect all dose
levels used
yall                <-list()                                                    # Summary vector to collect all
responses
ergall              <-list()                                                    # Object to collect all the model fits
sig<-array(0,k)
for (i in 1:(k))                                                           {
  ind1              <-as.logical((dat$Ray==rays[i])+(dat$Ray==rays[8]))        # Pick one ray at a time, include DMSO in every ray
  x0                <-10^(dat$ConcA[ind1])+10^(dat$ConcB[ind1])                 # Extract corresponding data for total
dose from data set
  y0                <-dat$Effect[ind1]                                          # Extract corresponding responses from
data set
  erg               <-try(fit(x0,y0,x))                                         # Fit loglogistic function to ray data,
calculate parameter estimates and predictions
  sig[i]     <-sqrt(sum(((erg[[1]][[7]])[,2])^2)/(length(x0)-4))           # Calculate an estimate of the error standard deviation in this
ray
  xall              <-c(xall,list(x0))                                          # Write data and results into summayr
objects
  yall              <-c(yall,list(y0))
  ergall     <-c(ergall,list(erg))                                  }
sigma               <-sqrt(mean(sig^2))                                         # Average error standard deviation over
all rays

#png(file="DR Curves.png",width=900, height=720)

plot(log10(xall[[1]]),yall[[1]],xlab="Log Dose",ylab="Effect",col="2",xlim=c(-6,11),ylim=c(0,1.1))
lines(log10(x),ergall[[1]][[3]],col="2")                                   # Plot dose response functions for all rays
for (i in 2:k)                                                                      {
  points(log10(xall[[i]]),yall[[i]],col=i+1)                               # Actual data points
  lines(log10(x),ergall[[i]][[3]],col=i+1)                          }      # fitted curves

legend(7.5, 1.1, legend=rays[1:k],col=c(2:(k+1)),lty=1, cex=0.8)

#dev.off()

################### Calculate EDp levels and taus at given effect levels y
##########################################################################

offset              <-10                                                                   # Offset is needed for
technical reasons, as control is not always exactly 100% response
taugrid             <-c(1:(2000-offset))/2000                                   # Dense grid of possible response levels
between 0 and approximately 1 (in fact, 1-Offset/2000) to calculate corresponding dose for
y                   <-c(0.1,0.2,0.3,0.4,0.5,0.6,0.7,0.8,0.9)                   # Smaller grid to be used for output

edall               <-array(0,c(k,length(taugrid)))                             # Summary object to collect all the EDp
levels
colnames(edall)     <-taugrid
rownames(edall)     <-rays[1:k]
parameters          <-NULL

for(i in 1:k)                                                               {          # Calculate EDp levels and
extract parameter estimates for all rays
  para              <-ergall[[i]][[4]][,c(1,2,4)]                               # Extract parameters estimates, SDs and
p-values
  edall[i,]   <-inversefunk(para[c(2,3,1,4),1],taugrid)                    # use parameters to calculate dose needed to reach every
effect in taugrid
  parameters        <-rbind(parameters,para)                               }

parameters<-data.frame(rep(rays[1:k],each=4),rep(c("b","c","d","e"),k),round(parameters,4)) # collect parameters in table with labels
```

```
colnames(parameters)<-c("Ray","Parameter","Estimate","SE","p-value")
rownames(parameters)<-c()
# write.csv(parameters[,1:4],'parameters1.csv')                                    # Write parameter estimates table in an
external file

tauall<-rbind(rep(1,length(taugrid)),rep(1,length(taugrid)))                        # Vector to collect all combination indices tau for
values in taugrid
for (i in 3:k)                                                                      {
 tau                 <-faktor[i]*edall[i,]/edall[1,]+(1-faktor[i])*edall[i,]/edall[2,] # Calculate taus for each ray, according to formula
given in Lee et al., 2007
 tauall      <-rbind(tauall,tau)                                  }
rownames(tauall)<-rays[1:k]
taualldez  <-round(tauall[,round((length(taugrid)+offset)*y)],3)               # Select subgroup of only those combination indices
corresponding to deciles.

##########  Isoboles and Average Taus
###############################################################################################################

e1                        <-ergall[[1]][[4]][4,c(1)]                                # ED50 parameter of first substance
e2                        <-ergall[[2]][[4]][4,c(1)]                                # ED50 parameter of second substance
mix1                      <-(faktor/e1) /  (faktor/e1+(1-faktor)/e2)          # Vector of effect standardized mixture rates
mix2                      <-cbind(tauall[,round((length(taugrid)+offset)*0.5)], tauall[,round((length(taugrid)+offset)*0.75)],
                           tauall[,round((length(taugrid)+offset)*0.25)])          # Combination indices at 25%, 50% and 75% effect
mix                       <-cbind(mix1,mix2)
mix                       <-mix[order(mix[,1]),]

#png(file="CIatIsoboles.png",width=900, height=720)
plot(mix[,1],mix[,2],type='l',ylim=c(0,max(mix)+0.5),col='red',xlab='Mixture proportion',ylab='Combination Index')
lines(mix[,1],mix[,3],col='green')
lines(mix[,1],mix[,4],col='blue')
legend(min(mix[,1]),max(mix)+0.5,legend=c("y=0.5","y=0.75","y=0.25"),col=c("red","green","blue"),lwd=1,cex=1)
#dev.off()

tauav                     <-round(c(exp(apply(log(taualldez),1,mean)),exp(mean(log(taualldez)[3:k,]))),3)
names(tauav)[k+1]    <-'Geometric Mean'
tauav
#write.csv(round(tauav[3:(k+1)],3),'AverageTau.csv')
```

Supplementary Figure Legends

Supplementary Figure S1. Dot box plots of genes that are significantly upregulated in both relapsed neuroblastoma and medulloblastoma cases compared to ependymoma and high-grade glioma from the INFORM program as measured by RNA sequencing (log2-fold-change expression). ANOVA: analysis of variance, EPN: ependymoma, HGG: high-grade glioma, MB: medulloblastoma, NB: neuroblastoma.

Supplementary Figure S2. Comparison of RA responsiveness as measured by absolute IC50 values of culture models compared to *MYC(N)* expression. Dot plots depicting absolute ATRA IC50 values and log2 *MYC/MYCN* expression of 4 neuroblastoma and 8 brain tumor culture models with linear regression analysis. Brain tumor gene expression profiles were extracted Affymetrix microarray data. Out of the N=12 brain tumor culture models two were excluded because RA IC50 values could not be calculated due to no dose-dependent response (ONS-76, SU-DIPG-13) and two were excluded due to missing expression data (SJ-GBM-2, KNS-42). Neuroblastoma gene expression is derived from RNA sequencing. As no SK-N-BE(2)-C expression was available in this dataset, the parental cell line SK-N-BE(2) was chosen as a surrogate. CNS: brain tumor models, NB: neuroblastoma models.

Supplementary Figure S3. Median dcDSS values of all 76 library drugs for N=9 RA less-responsive models. Positive dcDSS values indicate beneficial (blue) and negative values indicate adverse (blue) combination effects. dcDSS: differential combination drug sensitivity score.

Supplementary Figure S4. Median dcDSS values of all 76 library drugs for N=7 RA sensitive models. Positive dcDSS values indicate beneficial (blue) and negative values indicate adverse (blue) combination effects. dcDSS: differential combination drug sensitivity score.

Supplementary Figure S5. Median dcDSS values of all 76 library drugs for N=3 MB_{G3} models. Positive dcDSS values indicate beneficial (blue) and negative values indicate adverse (blue) combination effects. MB_{G3}: Group 3 medulloblastoma. dcDSS: differential combination drug sensitivity score.

Supplementary Figure S6. Median dcDSS values of all 76 library drugs N=4 NB models. Positive dcDSS values indicate beneficial (blue) and negative values indicate adverse (blue) combination effects. dcDSS: differential combination drug sensitivity score.

Supplementary Figure S7. Median dcDSS values of all 76 library drugs for N=2 EPN models. Positive dcDSS values indicate beneficial (blue) and negative values indicate adverse (blue) combination effects. dcDSS: differential combination drug sensitivity score.

Supplementary Figure S8. Median dcDSS values of all 76 library drugs for N=6 HGG models. Positive dcDSS values indicate beneficial (blue) and negative values indicate adverse (blue) combination effects. dcDSS: differential combination drug sensitivity score.

Supplementary Figure S9. Retinoic acid/navitoclax cotreatment synergistically inhibits viability in culture models of both MB_{G3} and NB. The rays are illustrated as dose-response curves for the different combination ratios. The calculated concentration indices (CIs) are plotted depending on the effect (y = 0.75 means 75% viability) and mixture proportion as isobolograms. Synergy (as defined by CI < 0.9) for the RA/navitoclax combination was observed in cell lines of both entities, with the geometric mean of CIs lowest in HD-MB03 (mean CI = 0.415), while it was similar for D425 (mean CI = 0.833) and NB-S-124 (mean CI = 0.826). In particular, in D425 and NB-S-124, ATRA/navitoclax cotreatment effects were most synergistic at higher antitumoral activity, which was also observed in the matrix design calculations. **A:** Left: Dose-response curves from ATRA/navitoclax cotreatment 7 Ray design (red = 1 to 0, green = 0 to 1, blue = 4 to 1, gray = 1 to 4, turquoise = 1.86 to 1, yellow = 1 to 1.86, and pink = 1 to 1) after 144 h of treatment. Right: Corresponding concentration index (CI) isobolograms depending on mixture proportion and treatment effect (red = 0.5/50% viability, green = 0.75/75% vibility, blue = 0.25/25% viability) in two MBG3 cell cultures. Mean CI = geometric mean of CI values across mixture proportions and effect sizes for the corresponding culture model. Interpretation is as follows: CI > 1.1 = antagonism, CI < 1.1 to > 0.9 = additivity, CI < 0.9 = synergy. N=3. **B:** Dose-response curves and CI isobolograms from ATRA/navitoclax cotreatment 7 Ray design in two NB cell cultures after 144 h of treatment. N=3.

Supplementary Figure S10. Images of cellular morphology after prolonged RA/navitoclax treatment (crystal violet staining). HD-MB03 cells were treated for 8 d with DMSO control, 0.5 µM ATRA, 1 µM navitoclax, and the combination thereof. Each tile represents a random imaging site per technical replicate, thus displaying three images for each condition per independent experiment. Example: Tiles 2.1 are taken from the first technical replicate in independent experiment number 2. Scale bar: 100 µm.

Supplementary Figure S11. Images of cellular morphology after prolonged RA/navitoclax treatment (crystal violet staining). SK-N-BE(2)-C cells were treated for 8 d with DMSO control, 1 µM ATRA, 3 µM navitoclax, and the combination thereof. Each tile represents a random imaging site per technical replicate, thus displaying three images for each condition per independent experiment. Example: Tiles 2.1 are taken from the first technical replicate in independent experiment number 2. Scale bar: 100 µm.

Supplementary Figure S12. Images of cellular morphology after prolonged RA/navitoclax treatment (crystal violet staining). SJ-GBM-2 cells were treated for 8 d with DMSO control, 0.5 µM ATRA, 0.1 µM navitoclax, and the combination thereof. Each tile represents a random imaging site per technical replicate, thus displaying three images for each condition per independent experiment. Example: Tiles 2.1 are taken from the first technical replicate in independent experiment number 2. Scale bar: 100 µm.

Supplementary Figure S13. Images of cellular morphology after prolonged RA/navitoclax treatment (crystal violet staining). EPD210FH cells were treated for 8 d with DMSO control, 0.5 µM ATRA, 30 nM navitoclax, and the combination thereof. Each tile represents a random imaging site per technical replicate, thus displaying three images for each condition per independent experiment. Example: Tiles 2.1 are taken from the first technical replicate in independent experiment number 2. Scale bar: 100 µm.

Supplementary Figure S14. ATRA differentiating effects shift to increased induction of apoptosis in combination with navitoclax. Quantification of caspase-3 positive nuclei in % on images from high-content fluorescence microscopy of (**A**) Left: HD-MB03, (**B**) D425, (**C**) SK-N-

BE(2)-C and, (**D**) NB-S-124 after 72 h of treatment with DMSO control, 500/1000 nM ATRA (MB/NB), 100 - 3162 nM navitoclax, 10 - 316 nM entinostat (MB), 100 - 3162 nM i-BET151 (NB), and the respective combinations (green = single, yellow = ATRA combination). The large dots indicate the corresponding mean. **A**: Right: Quantification of fragmented nuclei in % on images from high-content fluorescence microscopy after 72 h of treatment with DMSO control, 500 nM ATRA, 100 - 3162 nM navitoclax, 10 - 316 nM entinostat, and the respective combinations (green = single, yellow = ATRA combination). The large dots indicate the corresponding mean. **E:** Mean (+/- SD) caspase-3/7 activity of HD-MB03 (above)/SK-N-BE(2)-C (below) after 18 h, 24 h, 48 h, 72 h, 96 h, and 144 h treatment with DMSO control (blue), 500/1000 nM ATRA (red), 1000/3000 nM navitoclax (green), and the respective combinations (yellow). The bars indicate the standard deviation. STS: staurosporine, death control.

Supplementary Figure S15. Influences of RA/navitoclax on cell death and clonogenic growth over time. **A:** Mean % of dead cells of HD-MB03 (left)/SK-N-BE(2)-C (right) after 18 h, 24 h, 48 h, 72 h, 96 h, and 144 h (just in HD-MB03) treatment with DMSO control (blue), 500/1000 nM ATRA (red), 1000/3000 nM navitoclax (green), and the respective combinations (yellow). The bars indicate the standard deviation. N = 3 **B:** Colony assays of HD-MB03 (above)/SK-N-BE(2)-C (below) after 96 h of treatment with DMSO control (blue), 500/1000 nM ATRA (red), 1000/3000 nM navitoclax (green), and the respective combinations (yellow), followed by drug removal and further culture for 7 d. Left: three representative wells per treatment condition, right: quantification of relative colony formation compared to DMSO control. The horizontal bars indicate the mean. N = 5 for HD-MB03, N = 3 for SK-N-BE(2)-C.

Supplementary Figure S16. High-content timelapse imaging of patient-derived tumoroid growth upon repeated treatment exposure. Violin plots depicting tumoroid size change from baseline (%) of **A:** Med_2112fh_PDX_FTC (MB_{G3}), **B:** INF_R_1632_relapse1_PDX_LTC (NB), and **C:** INF_R_1887_relapse1_LTC (EPN) treated with DMSO control (blue), indicated concentrations of ATRA (red), navitoclax (green), and the respective combinations (yellow) with images taken on days 2, 4, 7, 9, 11, and 16 of the treatment regimen. All treatments were applied on days 0 and 7, treatment removal was carried out on days 4 and 11. As exemplarily indicated in the ATRA graph on the right. Gray areas in **A** highlight the first 7 days of culture in

which MB_{G3} tumoroids maintained viability in DMSO solvent control as prolonged culture showed treatment-independent tumoroid disintegration. N = 10 images per treatment.

Supplementary Figure S17. A: Relative viability as determined by metabolic activity assay (compared to DMSO control) of RCMB28 MB_{G3} tumoroids treated for 144 h with DMSO control (blue), ATRA 500 nM (red), 100/250/500nM navitoclax (green), and the respective combination (yellow). The horizontal bars indicate the mean. **B:** Representative zebrafish embryo xenograft images before (Day1) and after 48h (Day3) of treatment and corresponding waterfall plots representing the change from baseline growth of individual NB-S-124 zebrafish embryo neuroblastoma xenografts treated with DMSO (blue), 500 nM isotretinoin (red), 10 µM navitoclax (green), and combination (yellow) for 48 h. Each bar represents a single xenograft tumor. DMSO, N=25; isotretinoin, N=14; navitoclax, N=28; combo, N=13. PD: progressive disease; SD: stable disease; PR: partial response, DCR: disease control rate.

Supplementary Figure S18. Dot box plots of log2 z-score expression of anti-apoptotic genes BCL2, BCL2L1, and MCL1 as measured by RNA sequencing from n = 2288 relapsed pediatric tumors from the INFORM study. Box plots depict selected entities of pediatric nervous system tumors. ACC: adrenocortical carcinoma, ALL: acute lymphoblastic leukemia, AML: acute myeloid leukemia, ATRT: atypical teratoid/rhabdoid tumor, brain_emb: non-ATRT/non-MB embryonal brain tumors, EPN: ependymomas, EWS: Ewing sarcoma, GCT: germ cell tumor, HB: hepatoblastoma, HCC: hepatocellular carcinoma, HGG: high-grade glioma, MB: medulloblastomas, MRT: malignant rhabdoid tumor, NB: neuroblastomas, NHL: non-Hodgkin lymphoma, OS: osteosarcoma, RMS: rhabdomyosarcoma, sarc_other: non-EWS/non-RMS sarcoma.

References

1 Milde, T., Lodrini, M., Savelyeva, L., Korshunov, A., Kool, M., Brueckner, L. M. *et al.* HD-MB03 is a novel Group 3 medulloblastoma model demonstrating sensitivity to histone deacetylase inhibitor treatment. *Journal of neuro-oncology* **110**, 335-348 (2012).

2 Milde, T., Kleber, S., Korshunov, A., Witt, H., Hielscher, T., Koch, P. *et al.* A novel human high-risk ependymoma stem cell model reveals the differentiation-inducing potential of the histone deacetylase inhibitor Vorinostat. *Acta neuropathologica* **122**, 637-650 (2011).

3 van Tilburg, C. M., Pfaff, E., Pajtler, K. W., Langenberg, K. P. S., Fiesel, P., Jones, B. C. *et al.* The Pediatric Precision Oncology INFORM Registry: Clinical Outcome and Benefit for Patients with Very High-Evidence Targets. *Cancer discovery* **11**, 2764-2779 (2021).

4 Muller, M., Rosch, L., Najafi, S., Gatzweiler, C., Ridinger, J., Gerloff, X. F. *et al.* Combining APR-246 and HDAC-Inhibitors: A Novel Targeted Treatment Option for Neuroblastoma. *Cancers (Basel)* **13** (2021).

5 Worst, B. C., van Tilburg, C. M., Balasubramanian, G. P., Fiesel, P., Witt, R., Freitag, A. *et al.* Next-generation personalised medicine for high-risk paediatric cancer patients - The INFORM pilot study. *European journal of cancer* **65**, 91-101 (2016).

6 Peterziel, H., Jamaladdin, N., ElHarouni, D., Gerloff, X. F., Herter, S., Fiesel, P. *et al.* Drug sensitivity profiling of 3D tumor tissue cultures in the pediatric precision oncology program INFORM. *NPJ Precis Oncol* **6**, 94 (2022).

7 ElHarouni, D., Berker, Y., Peterziel, H., Gopisetty, A., Turunen, L., Kreth, S. *et al.* iTReX: Interactive exploration of mono- and combination therapy dose response profiling data. *Pharmacol Res* **175**, 105996 (2022).

8 Gatzweiler, C., Ridinger, J., Herter, S., Gerloff, X. F., ElHarouni, D., Berker, Y. *et al.* Functional Therapeutic Target Validation Using Pediatric Zebrafish Xenograft Models. *Cancers (Basel)* **14** (2022).

9 Brabetz, S., Leary, S. E. S., Grobner, S. N., Nakamoto, M. W., Seker-Cin, H., Girard, E. J. *et al.* A biobank of patient-derived pediatric brain tumor models. *Nat Med* **24**, 1752-1761 (2018).

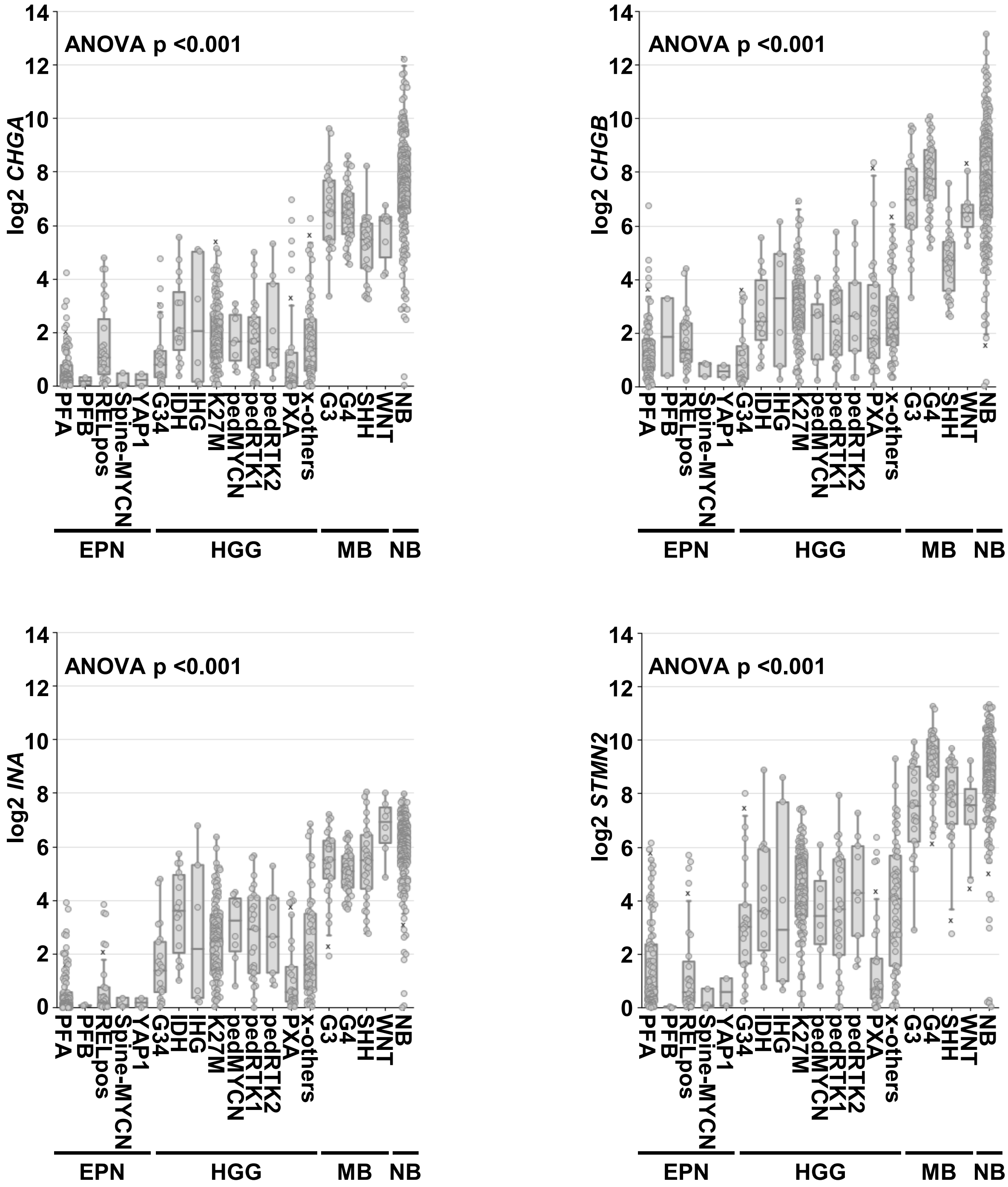

Suppl. Figure S1

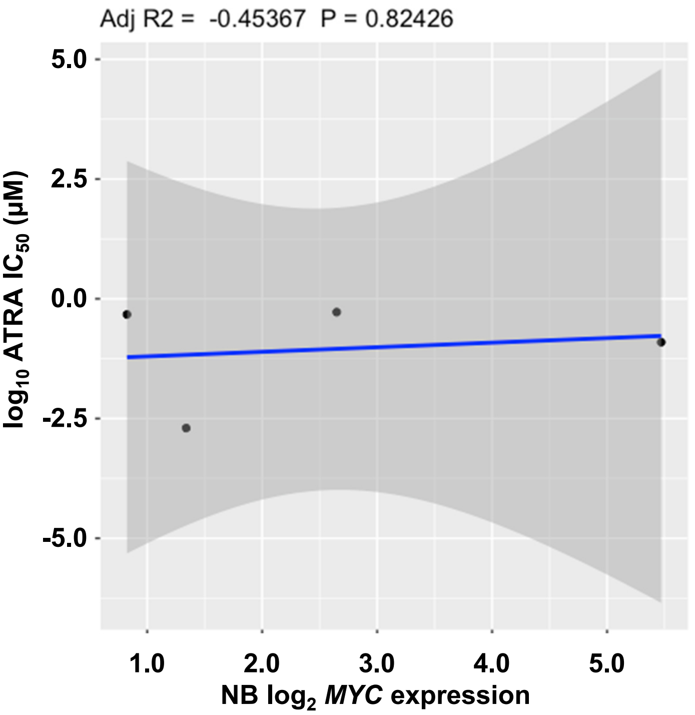

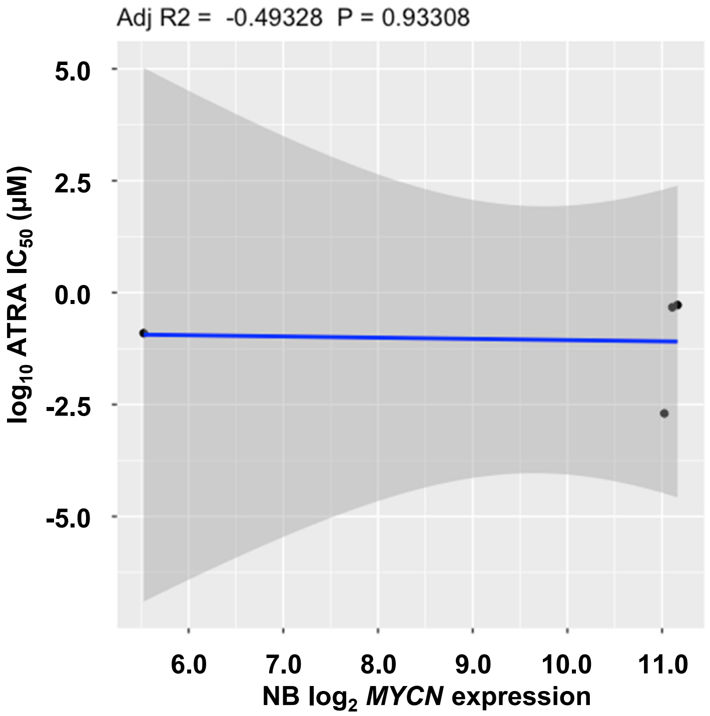

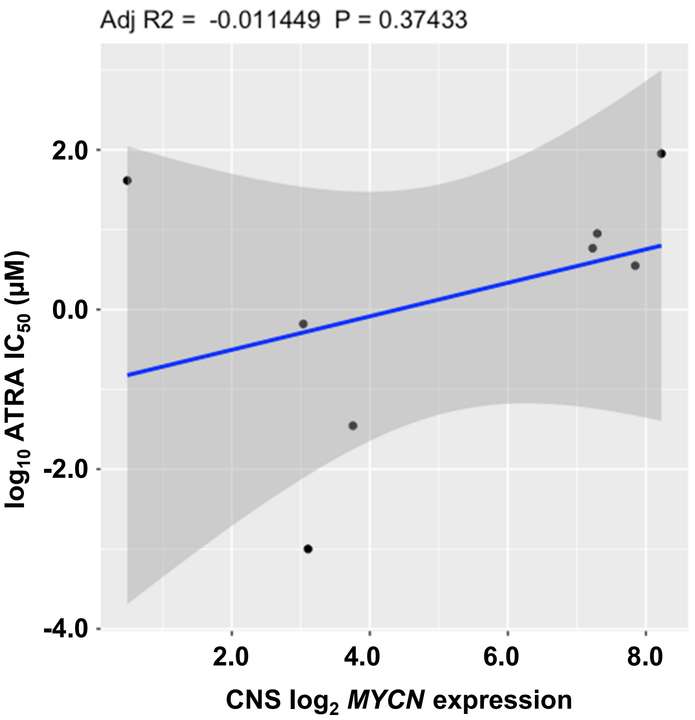

Suppl. Figure S2

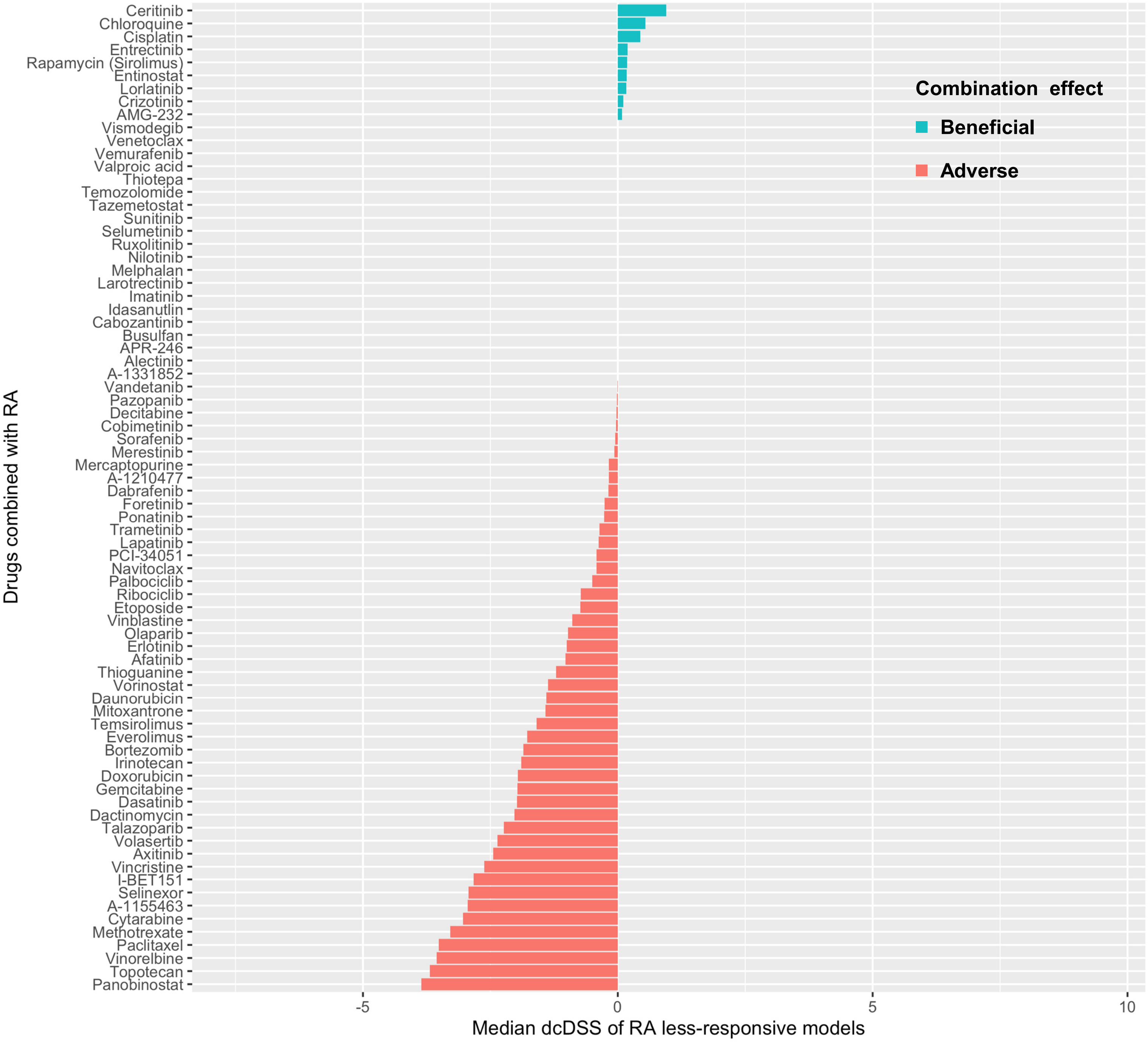

Suppl. Figure S3

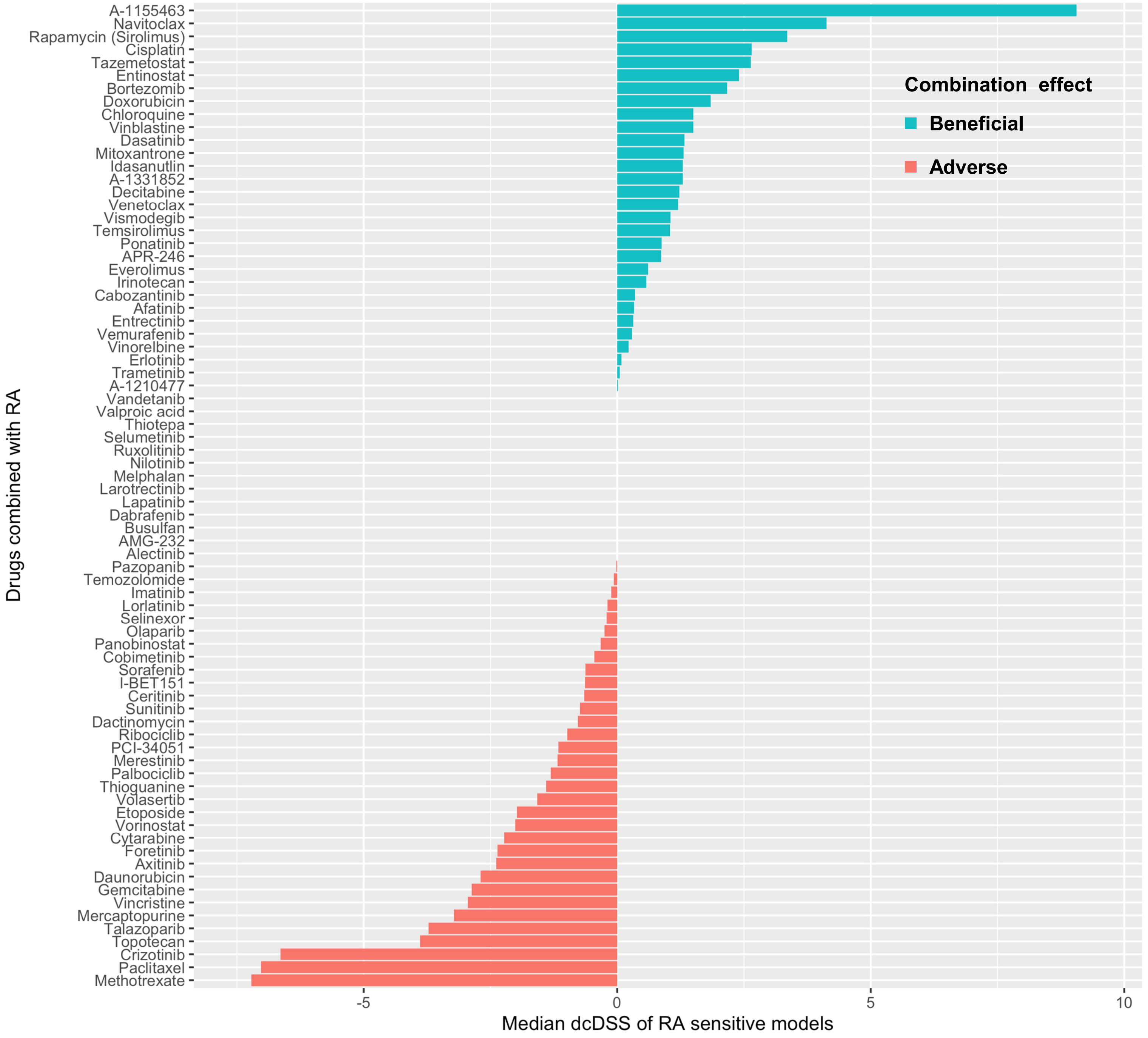

Suppl. Figure S4

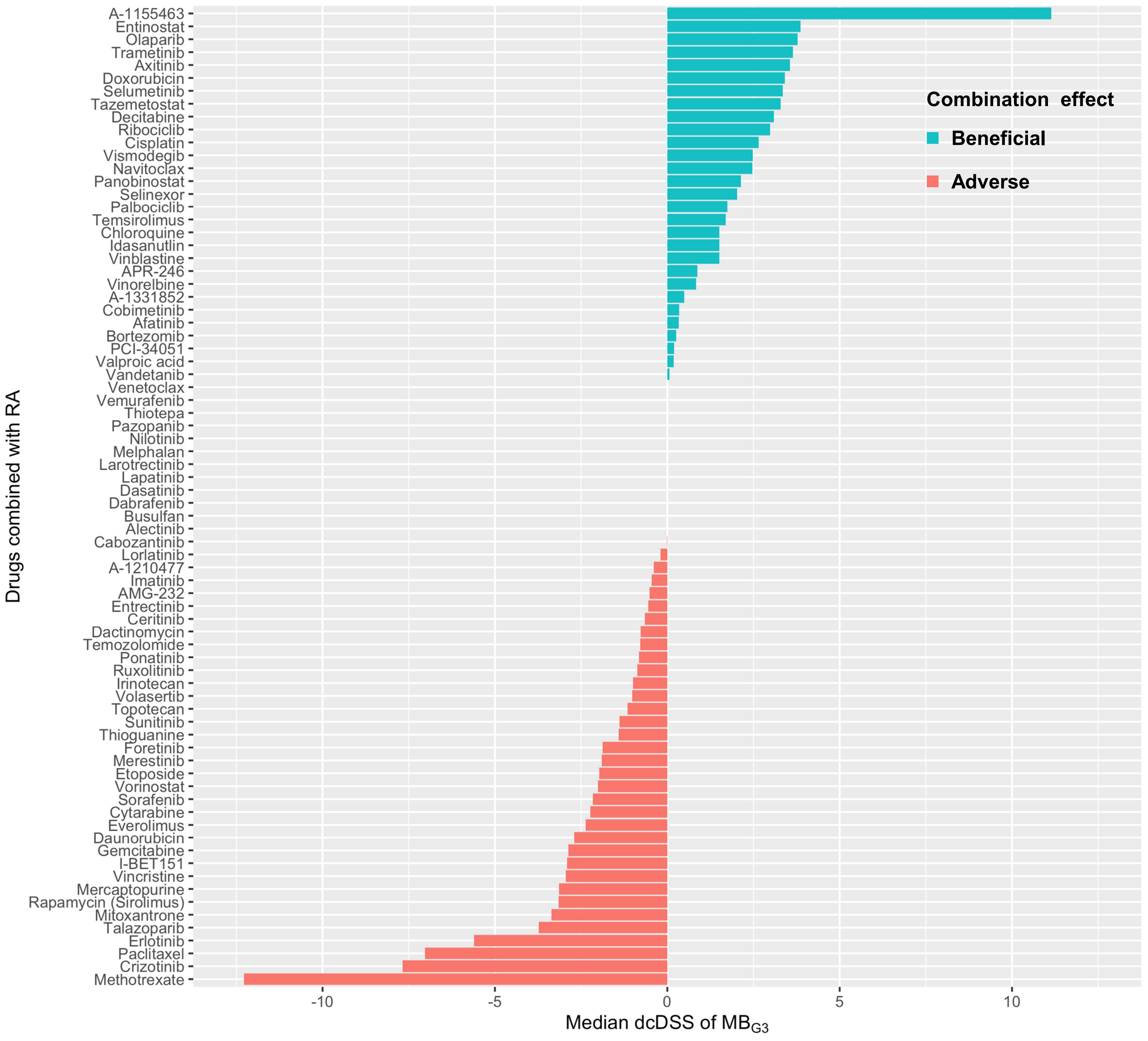

Suppl. Figure S5

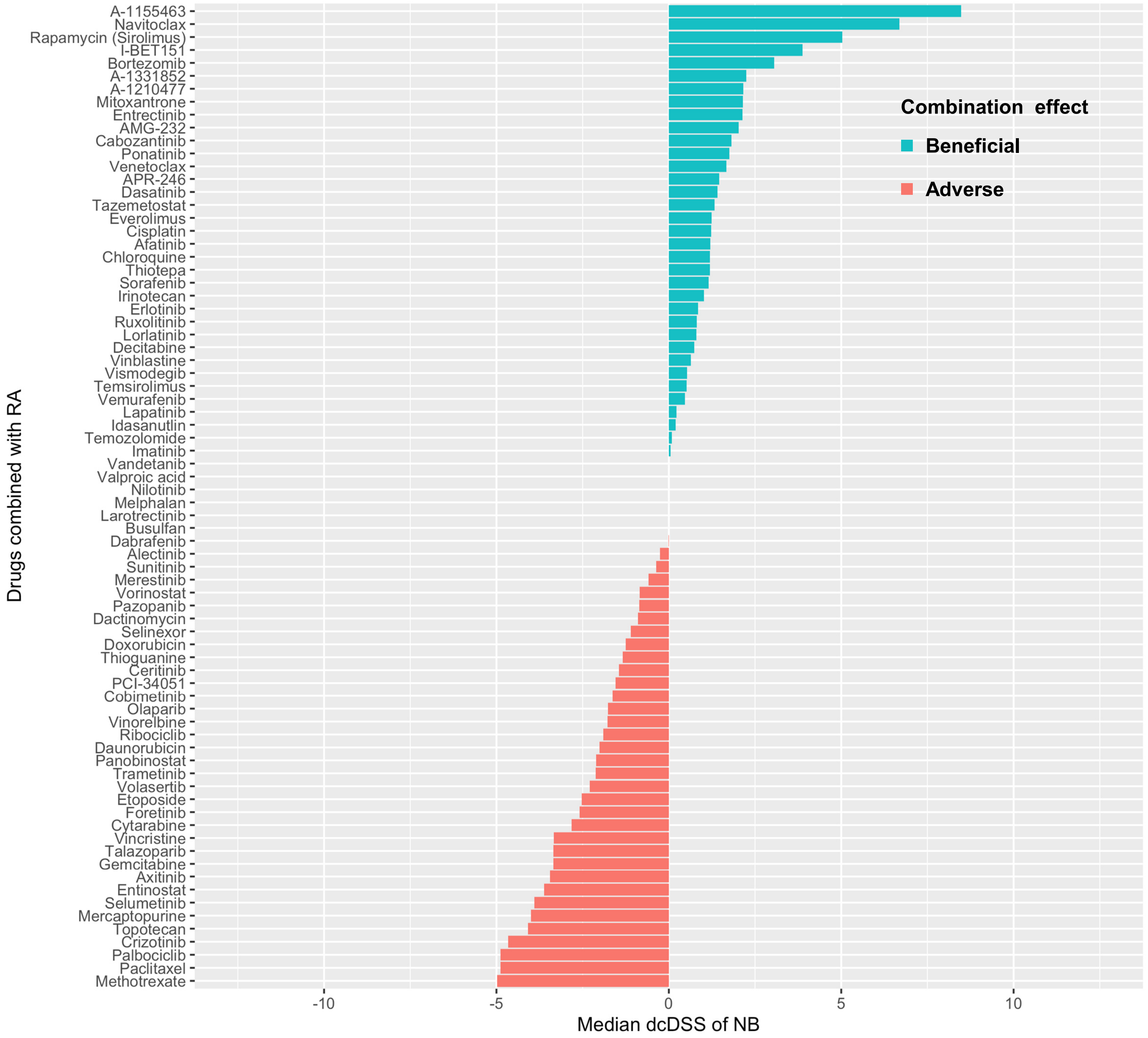

Suppl. Figure S6

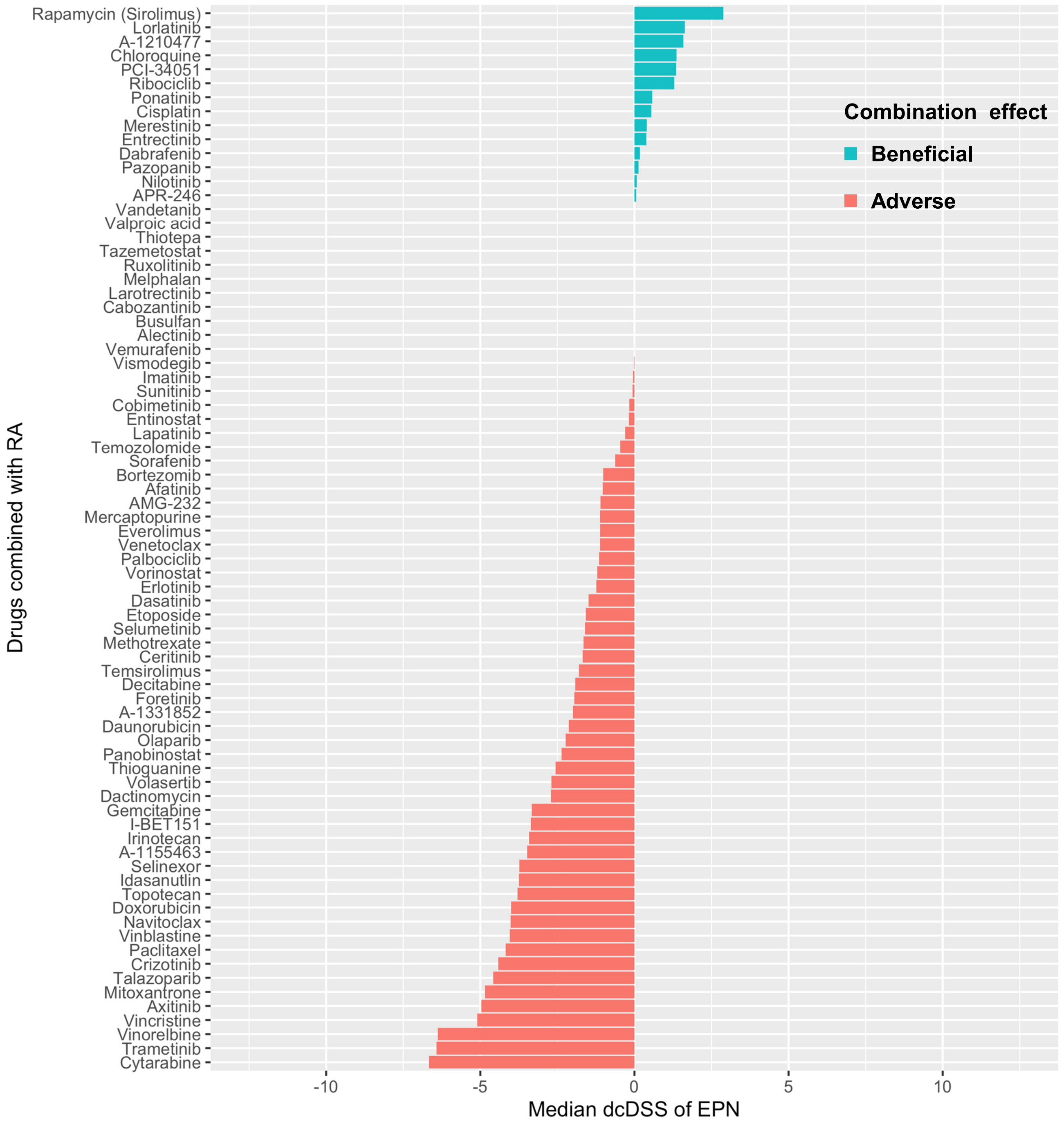

Suppl. Figure S7

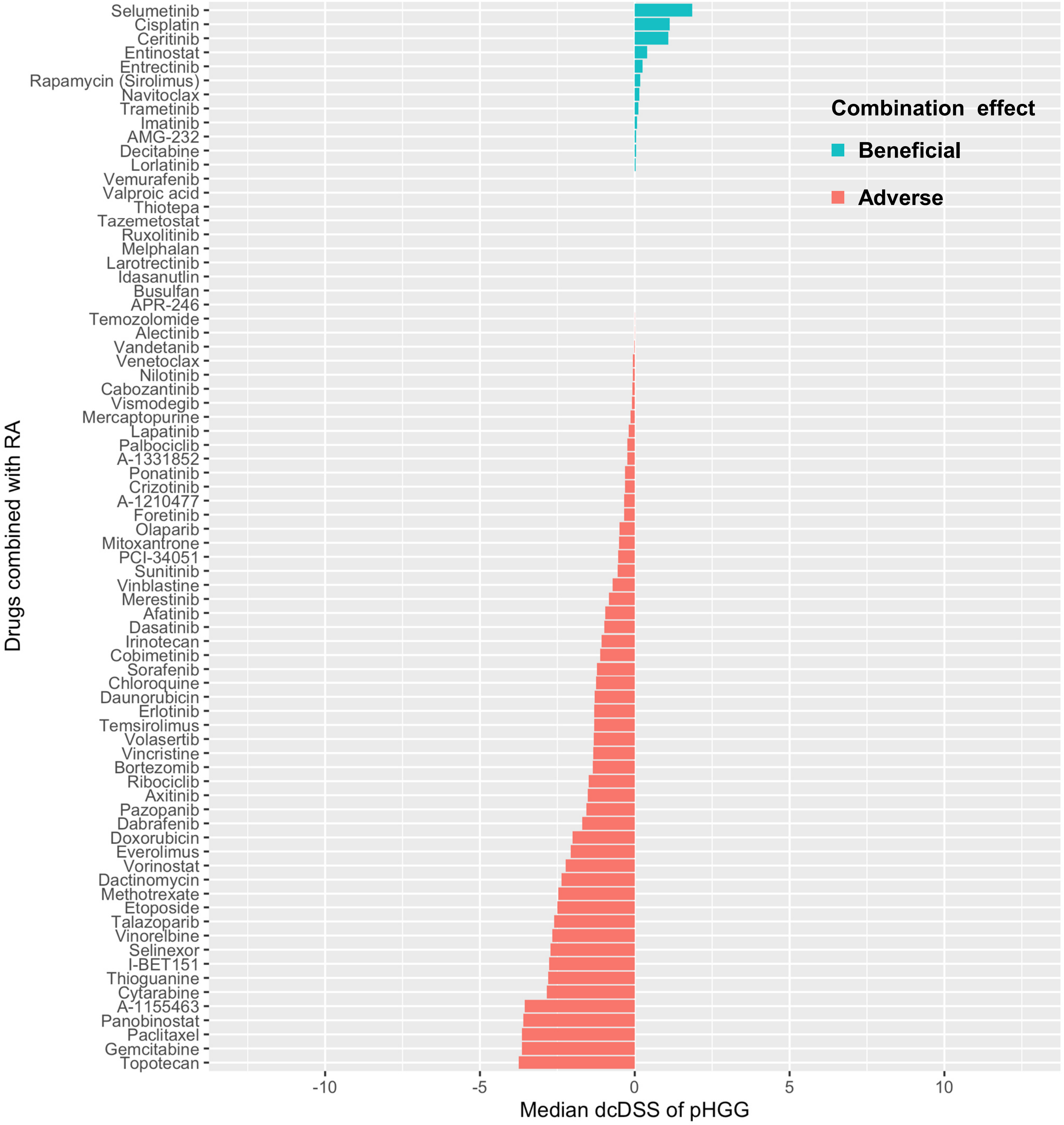

Suppl. Figure S8

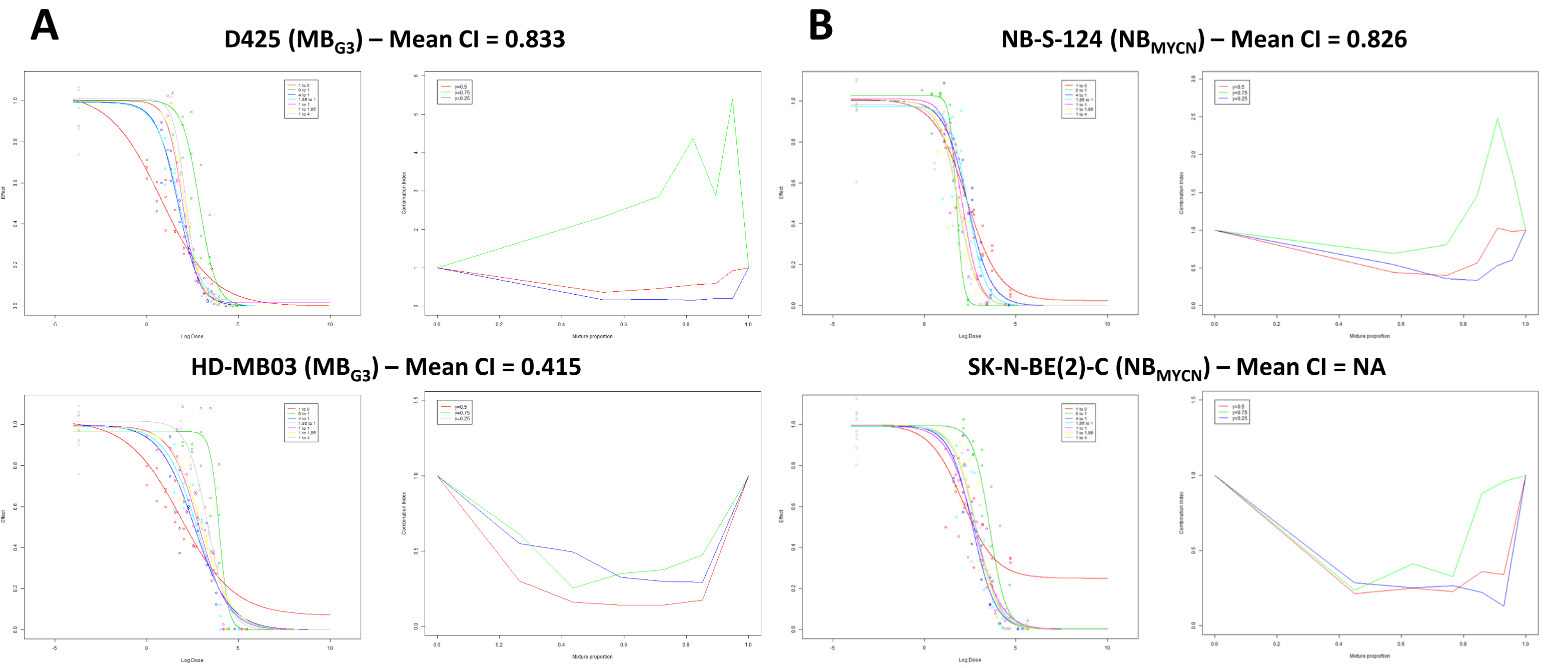

Suppl. Figure S9

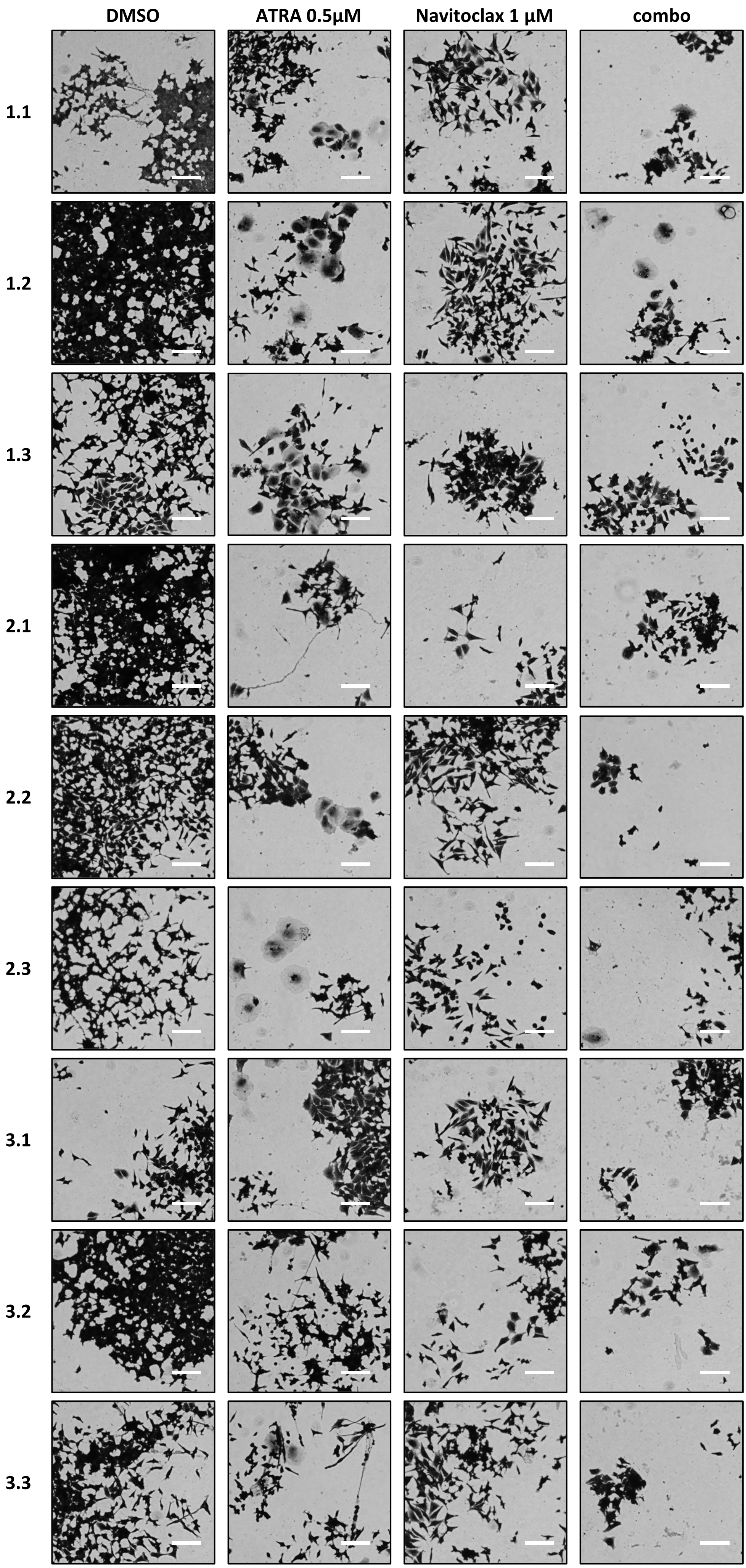

HD-MB03, 8 days treatment

Suppl. Figure S10

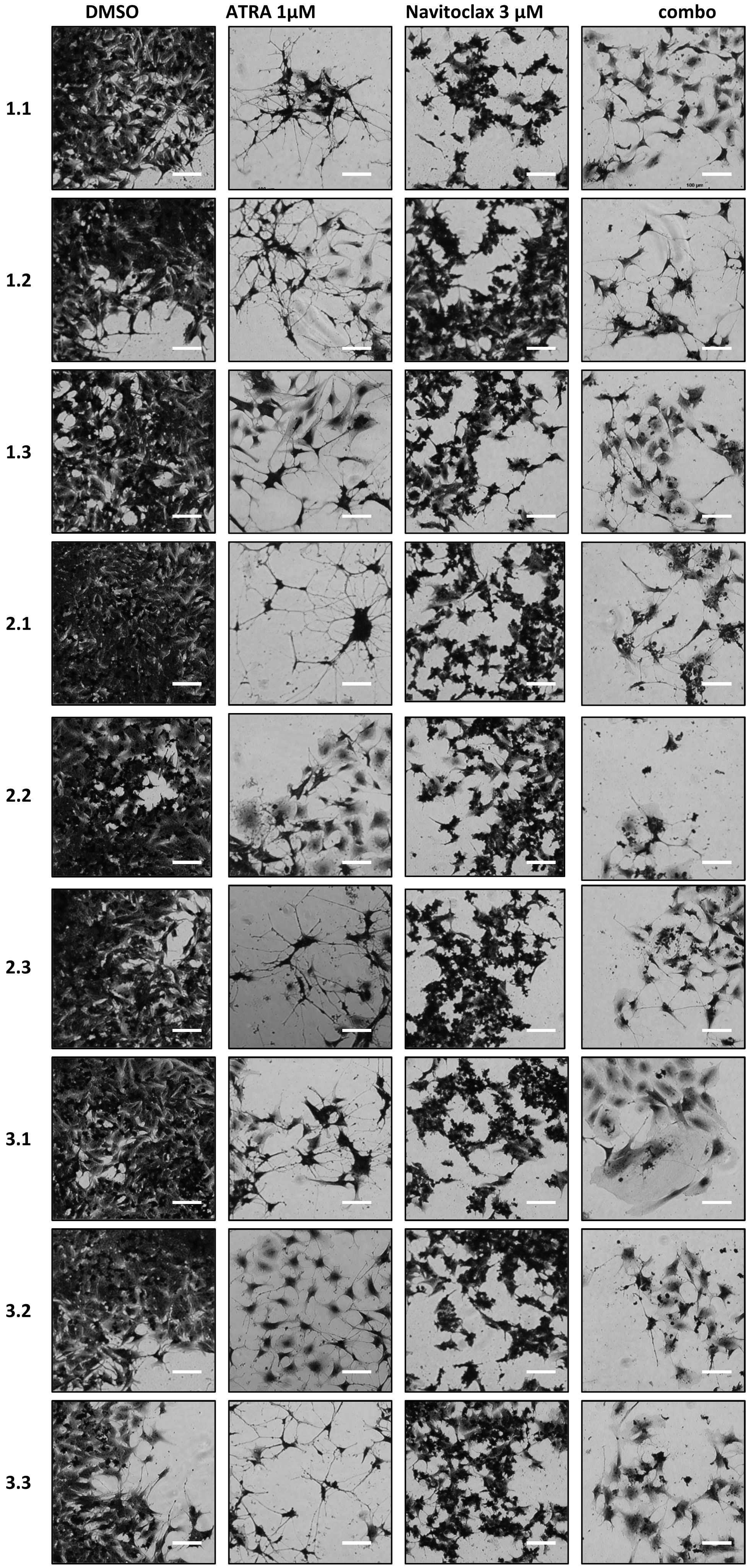

SK-N-BE(2)-C, 8 days treatment

Suppl. Figure S11

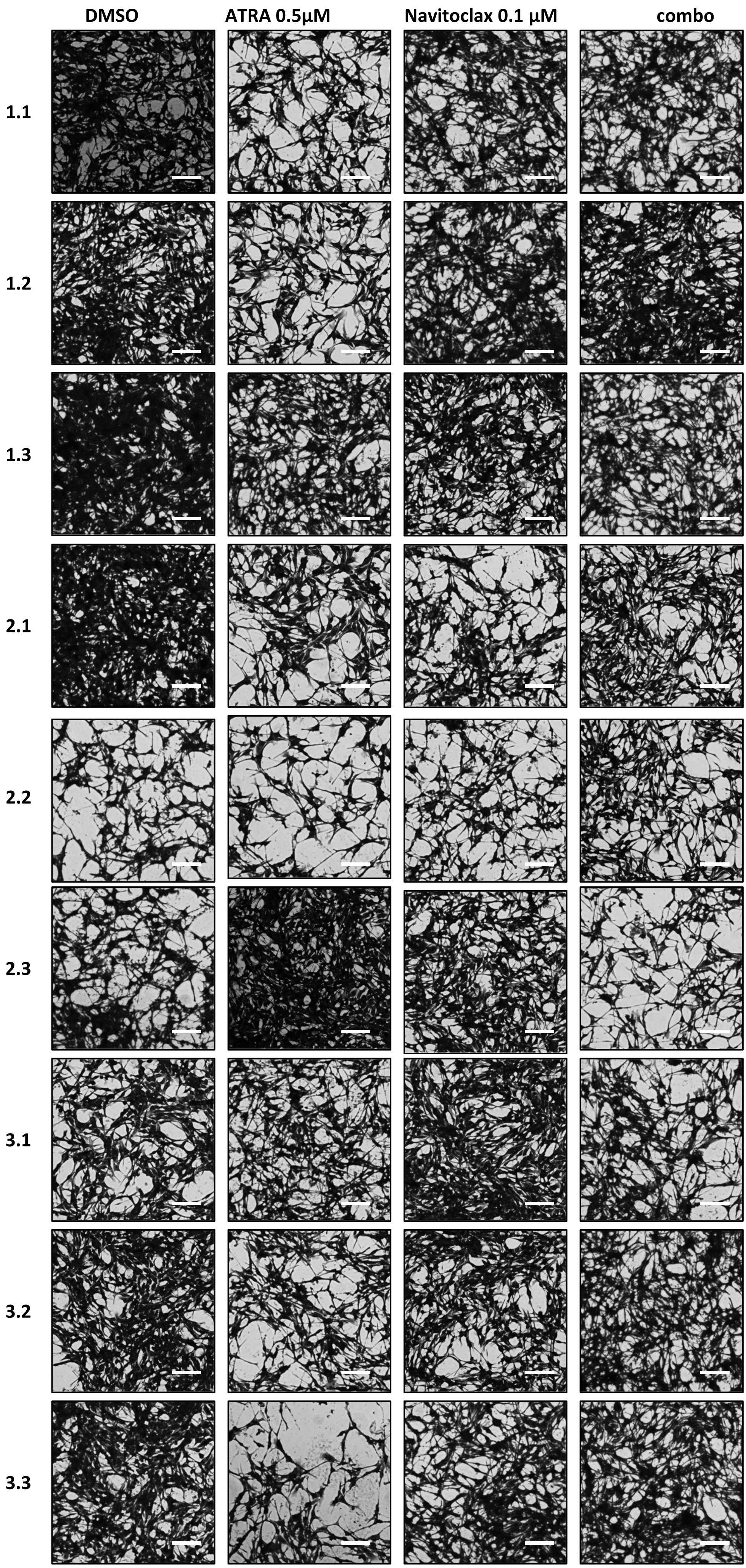

SJ-GBM2, 8 days treatment

Suppl. Figure S12

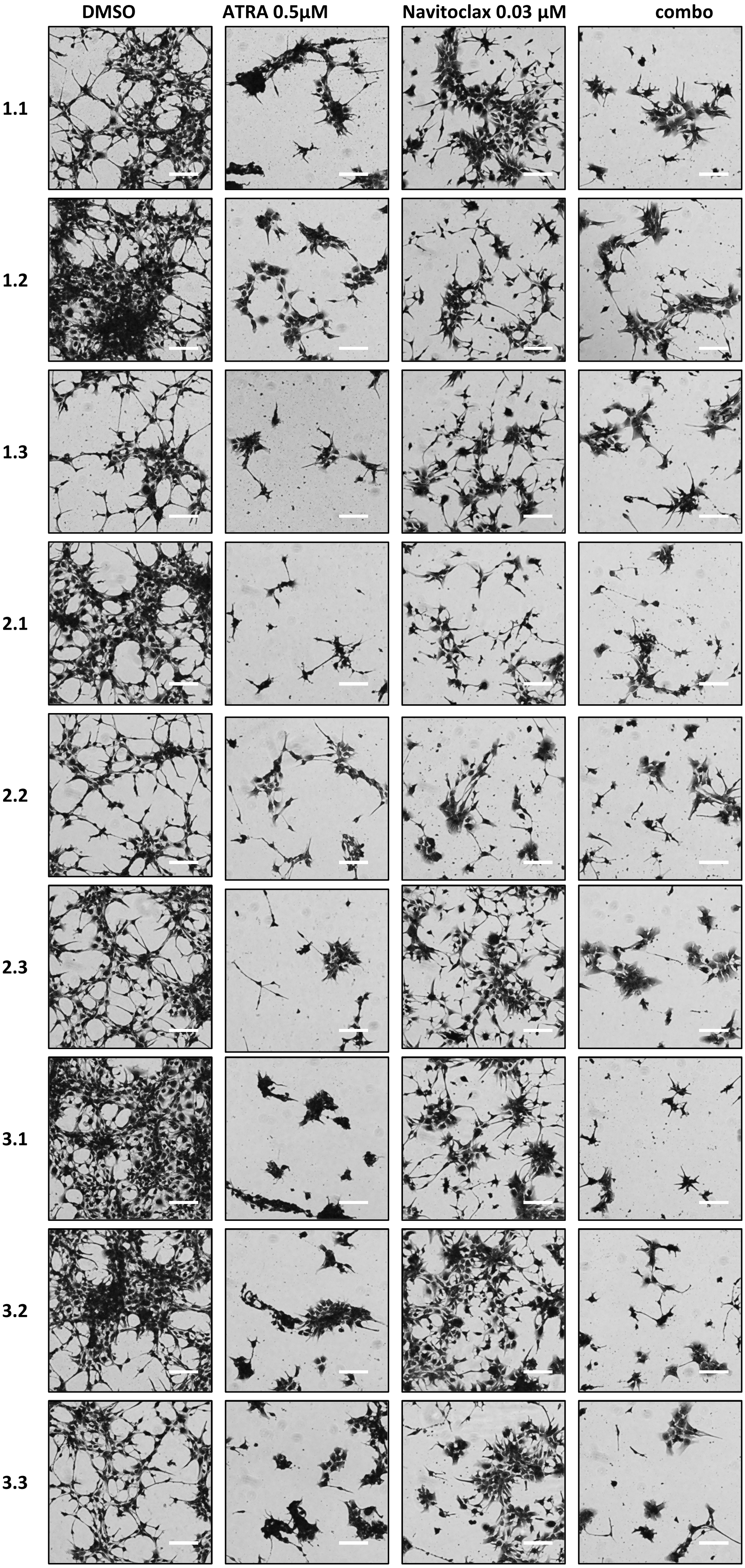

EPD210FH, 8 days treatment

Suppl. Figure S13

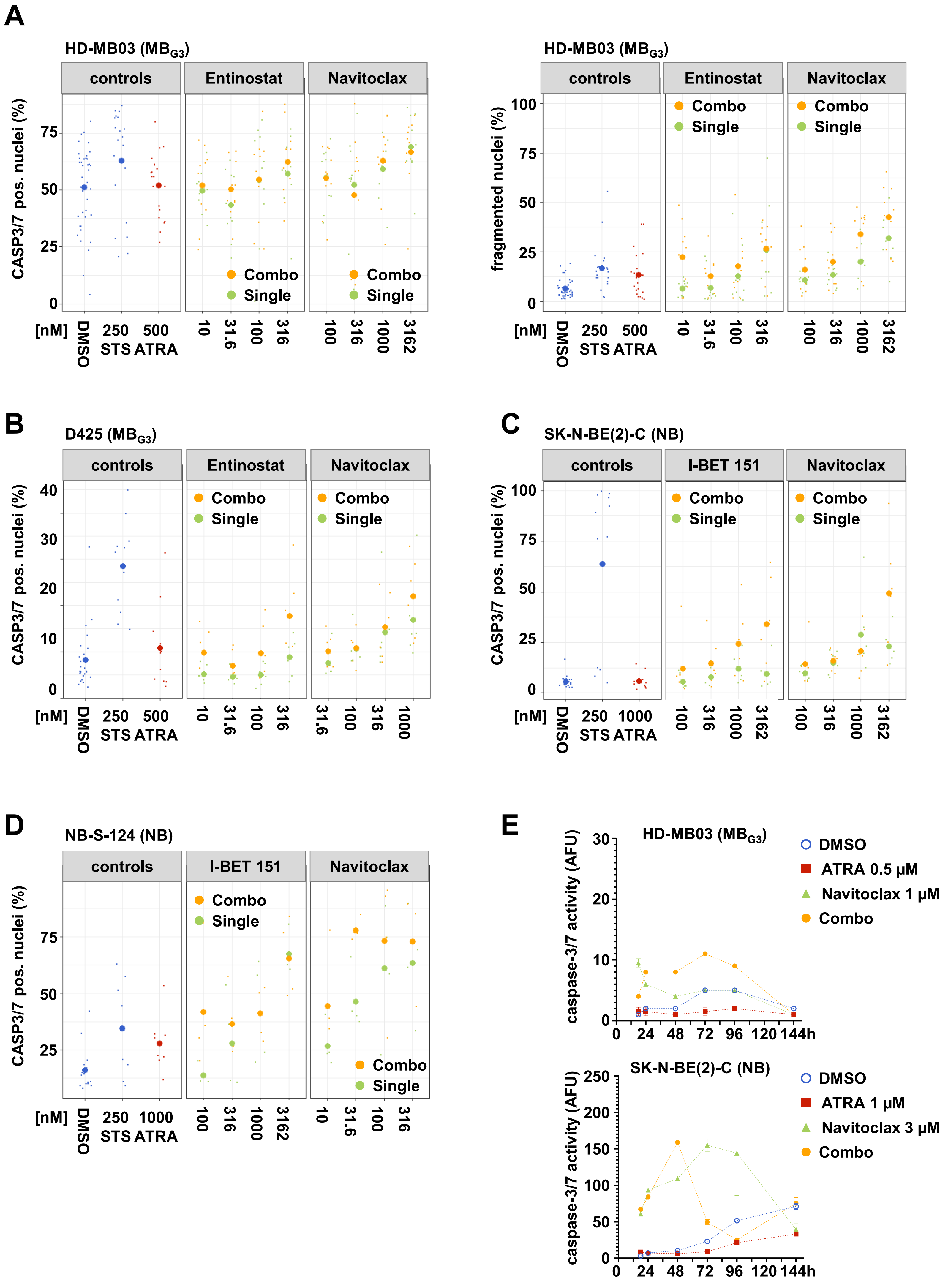

Suppl. Figure S14

A

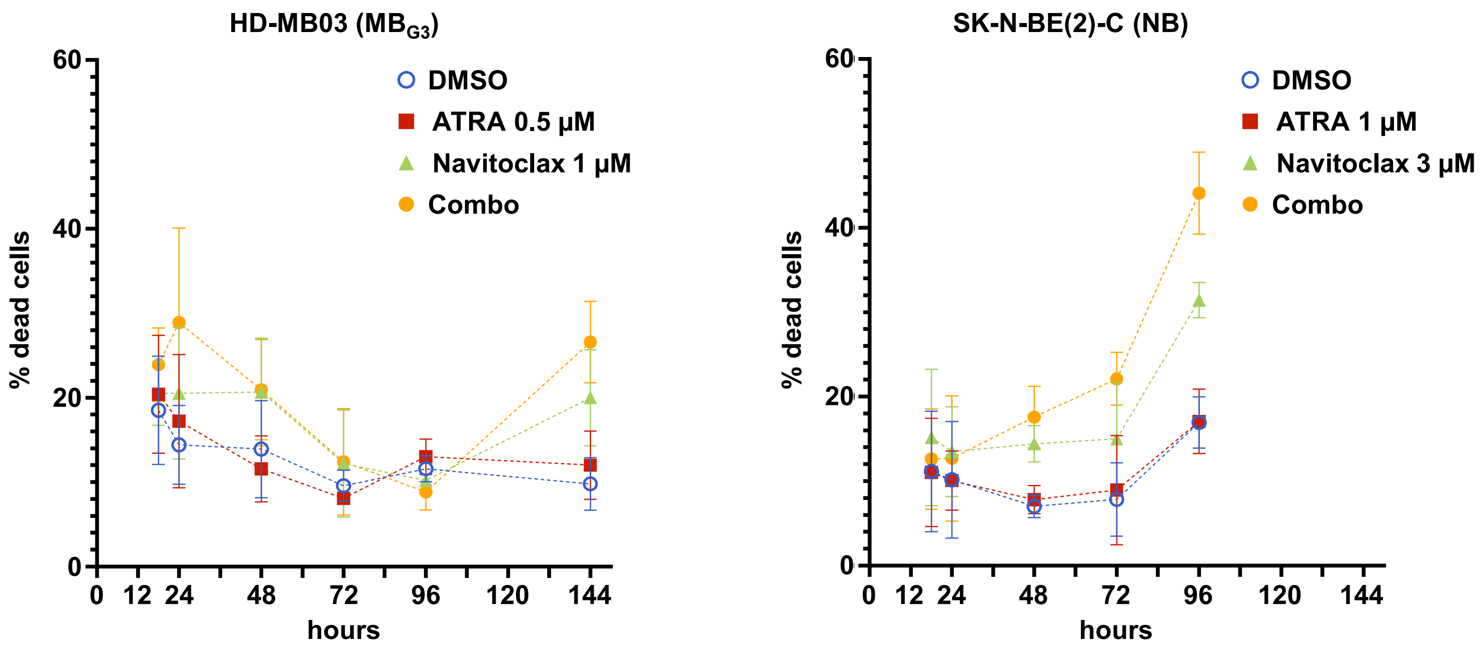

B

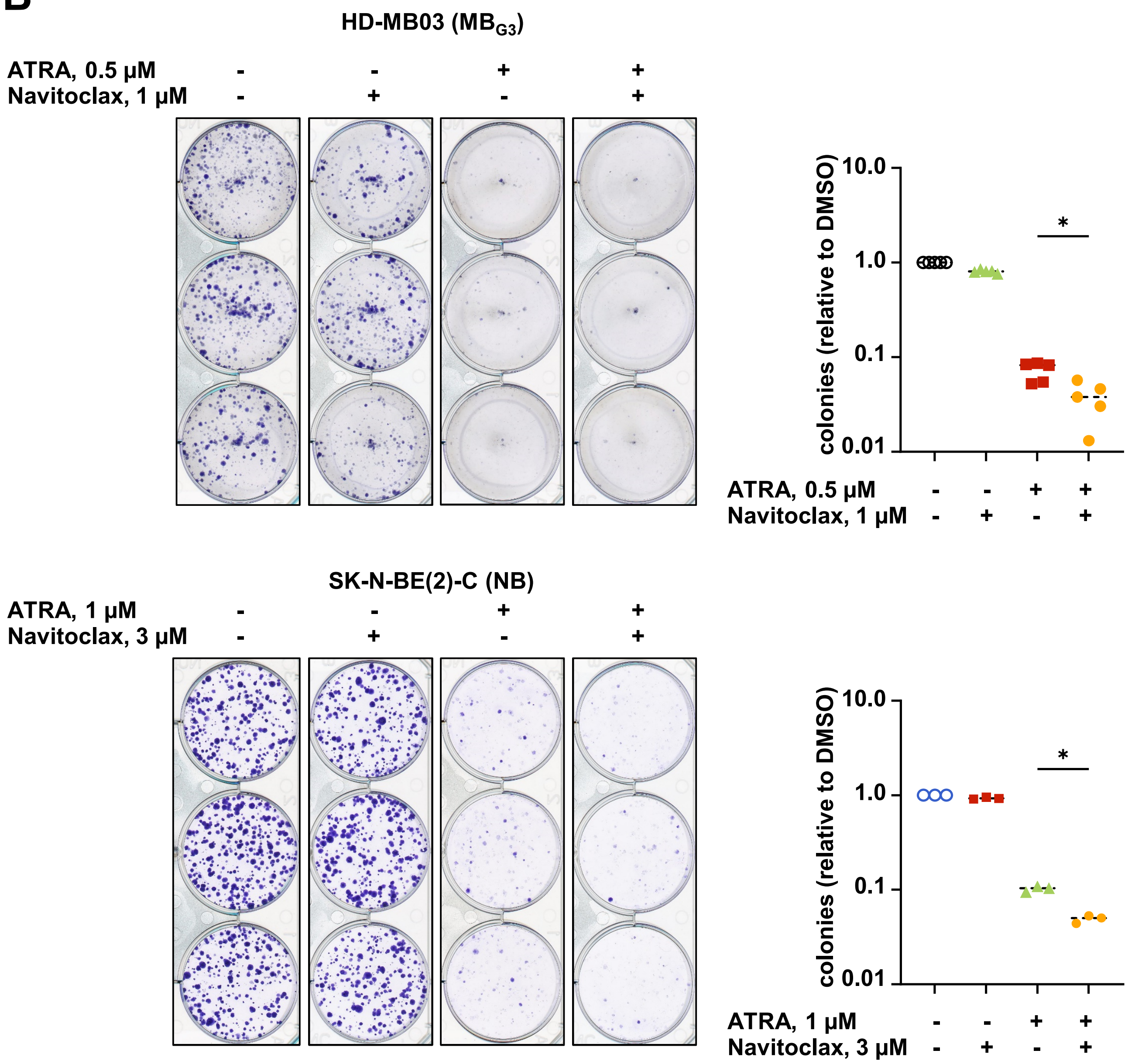

Suppl. Figure S15

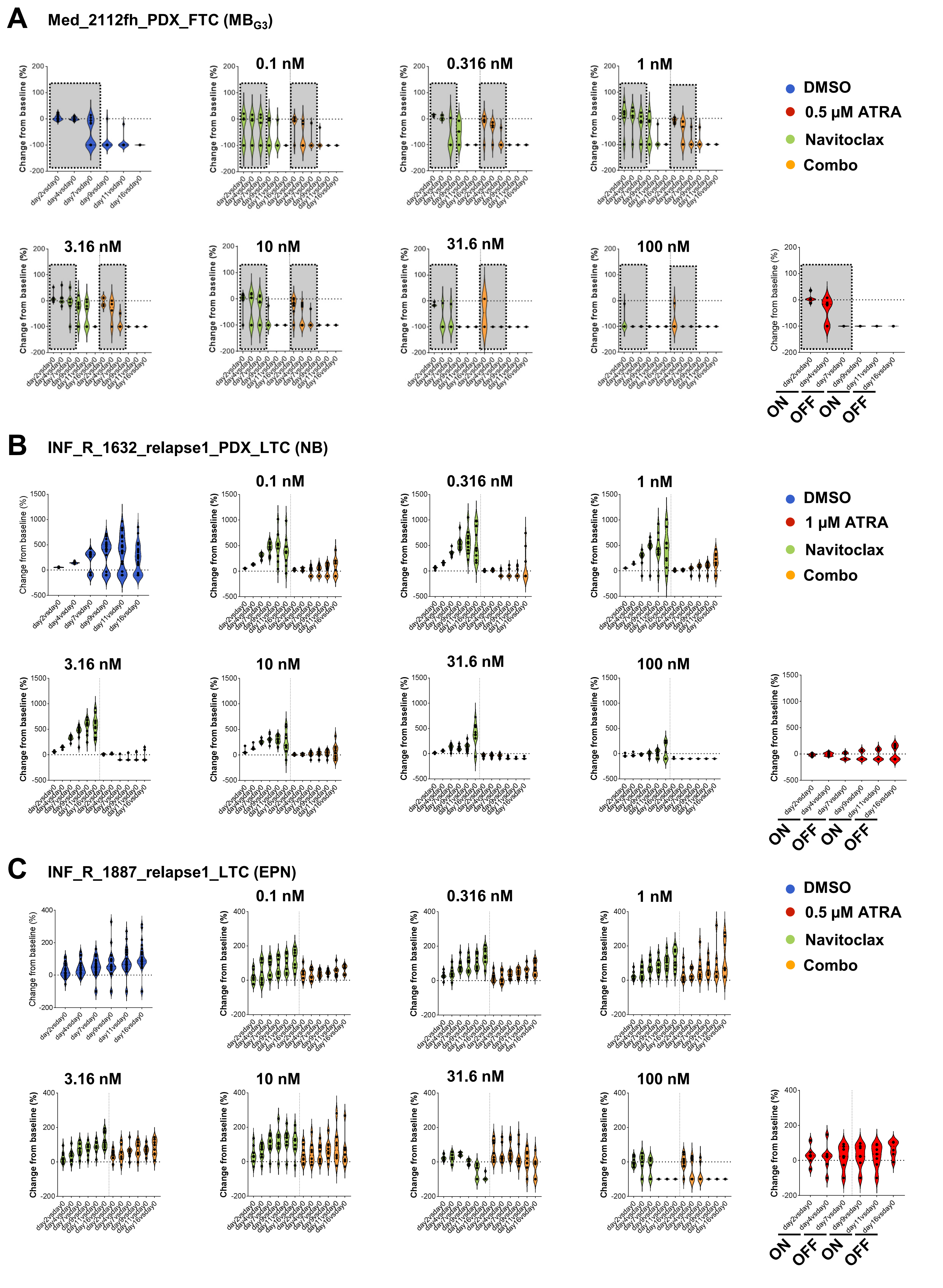

Suppl. Figure S16

A

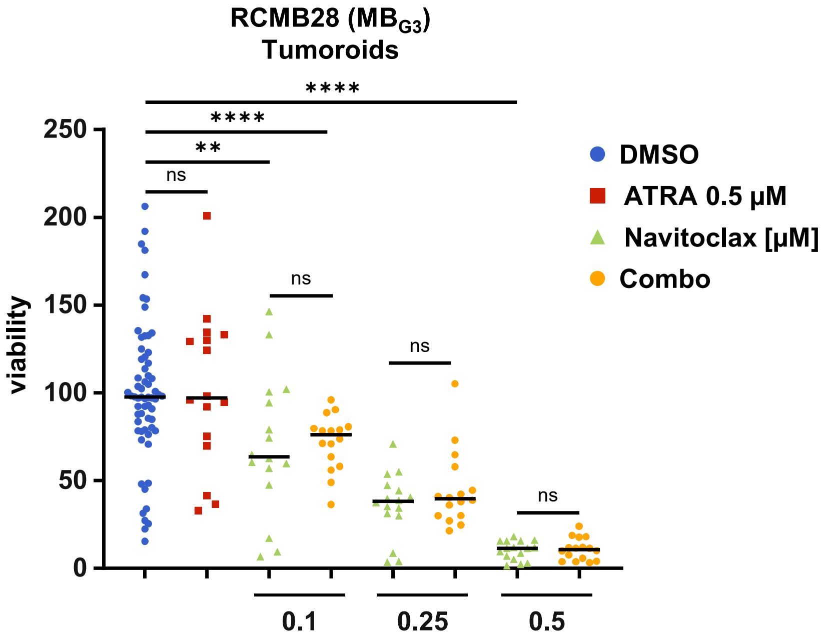

B

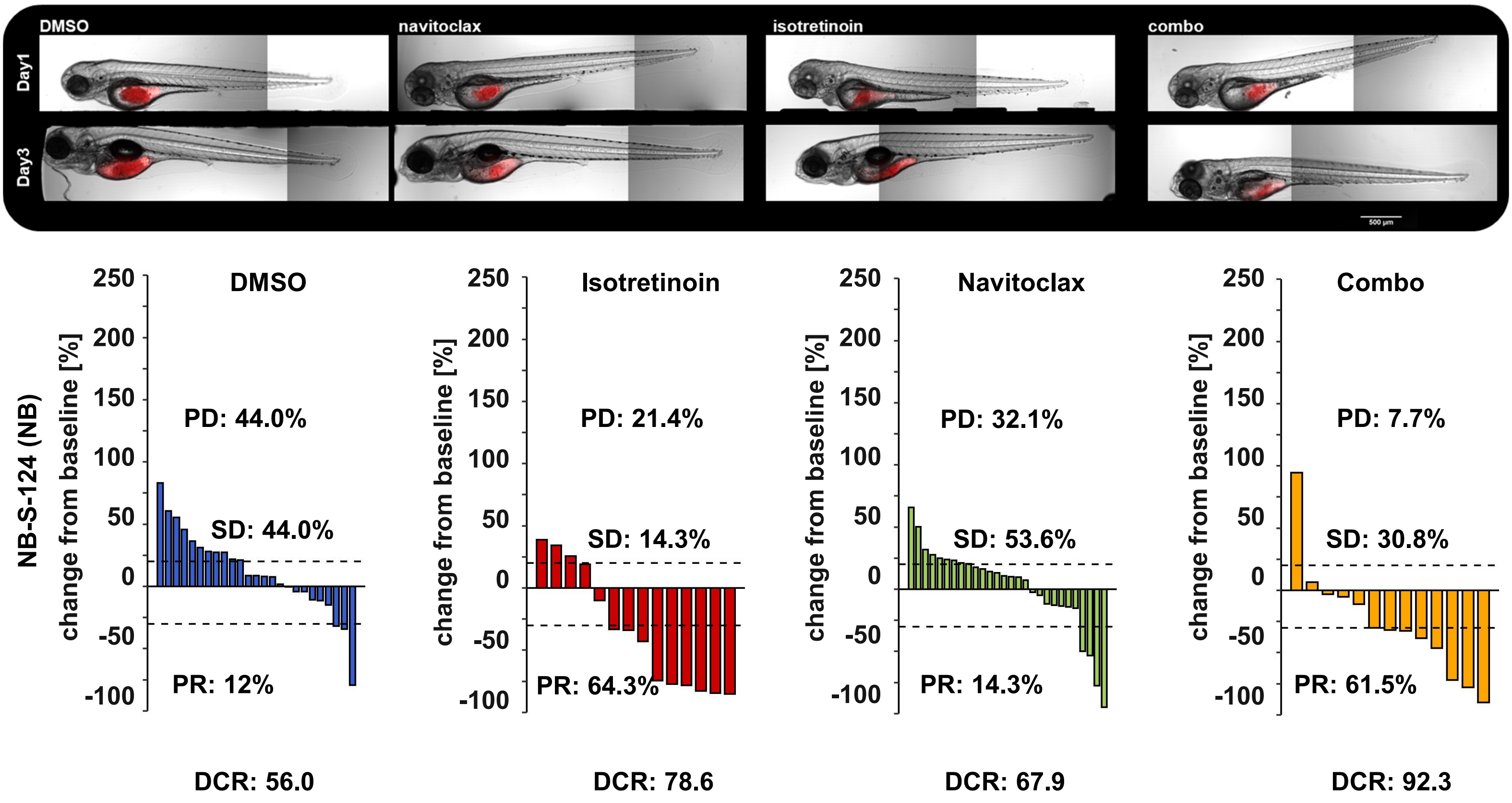

Suppl. Figure S17

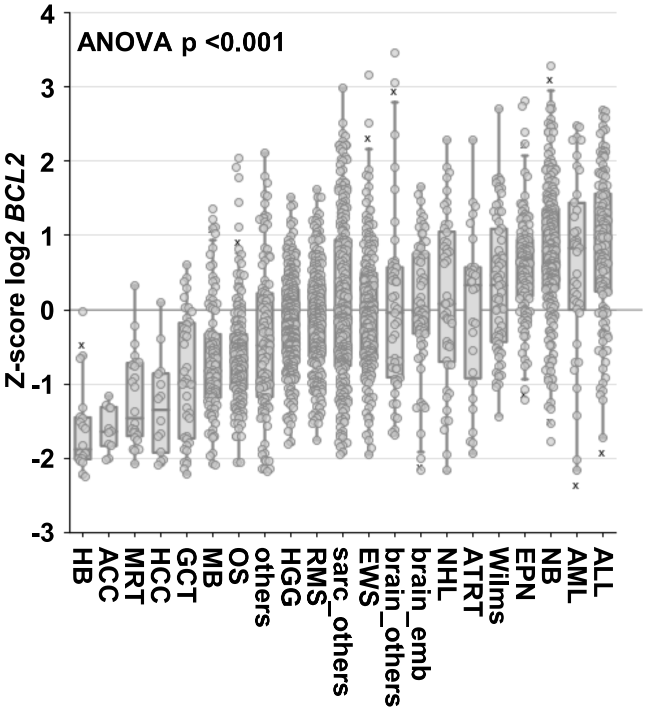

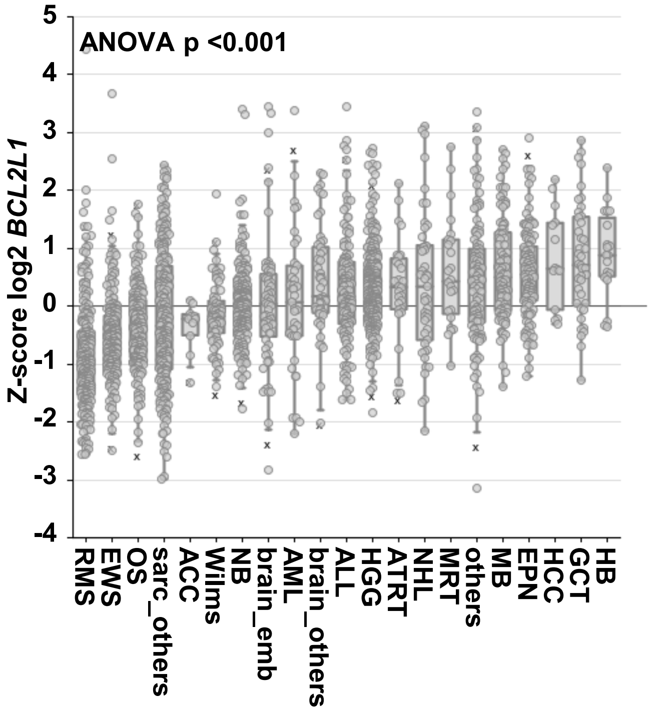

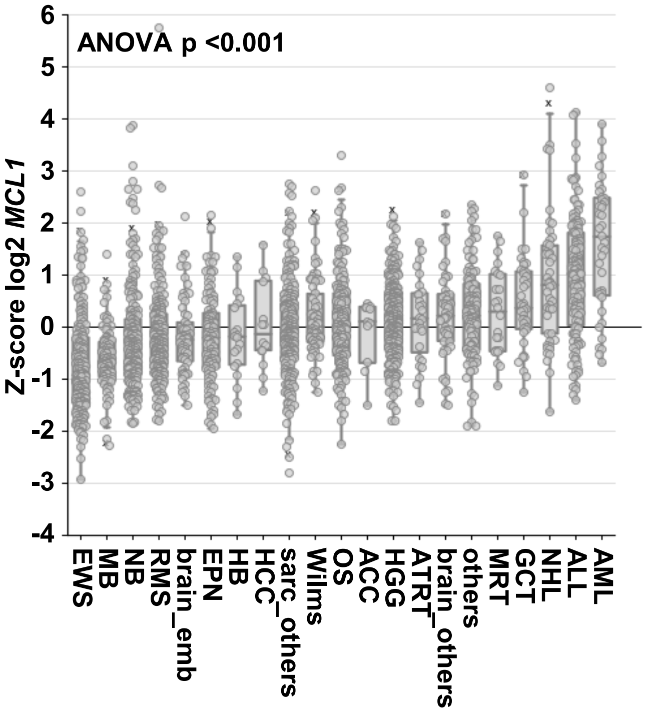

Suppl. Figure S18

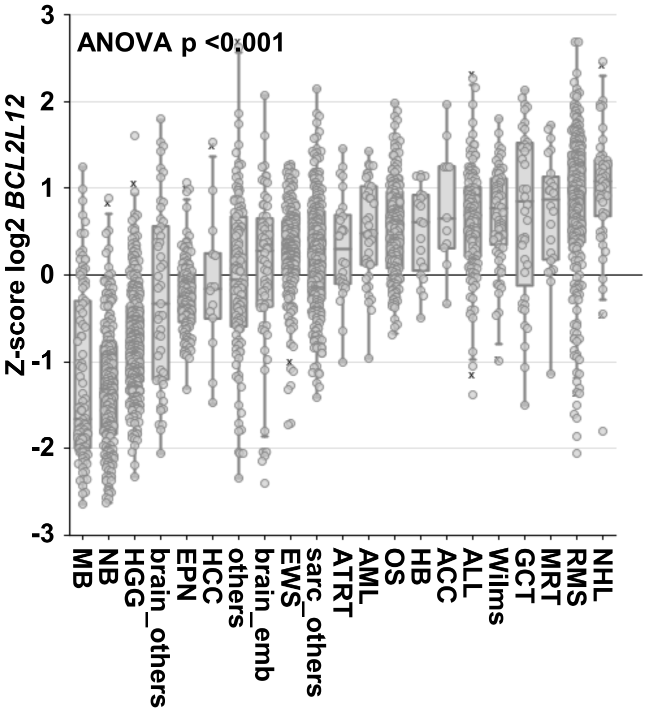

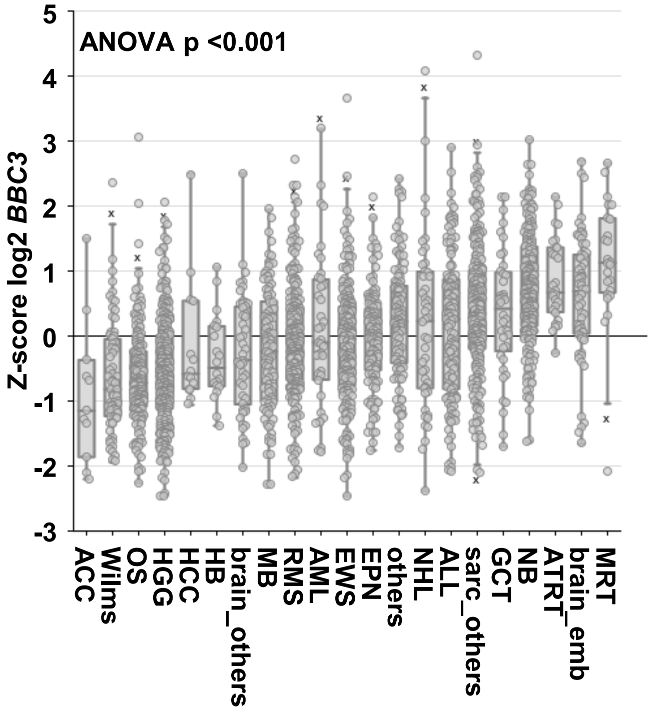

Supplementary Table 1: Fluorescence marker conditions for high content microscopy analysis.

**Marker**	**Supplier**	**Stained cell structure**	**Channel**	**Exposure time**	**Cat. No.**	**Concen-tration**
Hoechst33342	ThermoFisher Scientific	nucleic acid	DAPI	80 ms	H3570	1.11 µg/ml
CellEvent Caspase-3/7 Green	ThermoFisher Scientific	nucleic acid, indicating active caspases	FITC	50 ms	C10423	0.66 mM
CellMask Deep Red Plasma Membrane Stain	ThermoFisher Scientific	plasma membrane	Cy5	80 ms	C10046	2.22 µg/ml

Supplementary Table 2: CellProfiler pipeline and Cell Profiler Analyst settings used to identify Caspase positive cells. All non-mentioned settings were maintained in default mode.

**Order**	**Modul**	**Setting**
1	Smooth	Select input image: *Hoechst*<br>Select smoothing method: Median filter<br>Calculate artefact diameter automatically: Yes
2	IdentifyPrimaryObjects (nuclei)	Select input image: *Image generated by step 1*<br>Typical diameter of the object, in pixel units: 10 - 100<br>Discard object touching the border of the image: No<br>Thresholding method: Global Minimum Cross-Entropy<br>Threshold correction factor: 1.1<br>Lower and upper bounds on threshold: 0.0001 – 1.0<br>Method to distinguish clumped objects: Shape
3	MeasureObjectIntensity	Select images to measure: *Hoechst, Caspase*<br>Select objects to measure: *Objects identified in step 2*
4	MeasureObjectIntensity Distribution	Select images to measure: *Hoechst, Caspase*<br>Select objects to measure: *Objects identified in step 2*
5	MeasureGranularity	Select images to measure: *Hoechst, Caspase*<br>Measure within objects? Yes<br>Select objects to measure: *Objects identified in step 2*
6	MeasureObjectSizeShape	Select objects to measure: *Objects identified in step 2*
7	MeasureTexture	Select images to measure: *Hoechst, Caspase*<br>Measure whole images or objects? Both<br>Select objects to measure: *Objects identified in step 2*
8	ExporttoDatabase	Create a CellProfiler Analyst properties file? Yes<br>Which objects should be used for locations? *Objects identified in step 2*
9	CellProfiler Analyst	Open: *CellProfiler Analyst properties file generated by step 8*
10	Generating Classifier Training set	*Objects identified in step 2 r*andomly fetched from the whole data set; manually annotated into two classes, respectively: Caspase positive and negative nuclei or fragmented and healthy nuclei (n=1950)
11	Training Classifier for Caspase positive cells	Use: Random Forest Classifier<br>Classification accuracy: 95.69%<br>Five top features selected by program:<br>1) IdentifyPrimaryObjects_Hoechst_Intensity_MADIntensity_Caspase<br>2) IdentifyPrimaryObjects_Hoechst_RadialDistribution_MnFrc_Csps_4f4<br>3) IdentifyPrimaryObjects_Hoechst_Granularity_4_Caspase<br>4) IdentifyPrimaryObjects_Hoechst_Granularity_3_Caspase<br>5) IdentifyPrimaryObjects_Hoechst_Intensity_StdIntensity_Caspase
12	Training Classifier for Fragmented nuclei	Use: Random Forest Classifier<br>Classification accuracy: 98.00%<br>Five top features selected by program:<br>1) IdentifyPrimaryObjects_Hoechst_Texture_DffrncVrnc_Hchst_3_02_256<br>2) IdentifyPrimaryObjects_Hoechst_Intensity_StdIntensity_Hoechst<br>3) IdentifyPrimaryObjects_Hoechst_Texture_DffrncVrnc_Hchst_3_03_256<br>4) IdentifyPrimaryObjects_Hoechst_Granularity_1_Hoechst<br>5) IdentifyPrimaryObjects_Hoechst_Intensity_LowerQurtlIntnsty_Hchst
13	Classifier	Apply training sets to the whole data set; Export Hit Table (grouped by Image)

Supplementary Table 3: CellProfiler pipeline used to to quantify the length of cellular protrusions. All non-mentioned settings were maintained in default mode.

Order	Modul	Setting
1	Smooth	Select input image: *Hoechst*<br>Select smoothing method: Median filter<br>Calculate artefact diameter automatically: Yes
2	IdentifyPrimaryObjects (nuclei)	Select input image: *Image generated by step 1*<br>Typical diameter of the object, in pixel units: 10 - 100<br>Discard object touching the border of the image: No<br>Thresholding method: Global Minimum Cross-Entropy<br>Threshold correction factor: 1.1<br>Lower and upper bounds on threshold: 0.0001 – 1.0<br>Method to distinguish clumped objects: Shape
3	IdentifySecondaryObjects (soma)	Select input image: *CellMask*<br>Select the input objects: *Objects identified in step 2*<br>Select the method to identify the secondary object: Distance - B<br>Thresholding method: Global Otsu<br>2 classes or 3 classes : 3 classes<br>Assign pixel in the middle : foreground<br>Treshold correction factor: 1.1<br>Lower and upper bounds on threshold 0.00000001 – 1.0<br>Number of pixels by which to expand the primary objects? 20
**4**	EnhanceorSuppressFeatures	Select input image: *CellMask*<br>Select the operation: enhance<br>Feature type: neurite<br>Enhancement method: Line structures<br>Feature size : 15
**5**	Smooth	Select input image: *Hoechst*<br>Select smoothing method: Gaussian filter<br>Calculate artefact diameter automatically: No<br>Typical artificial diameter: 1
**6**	IdentifySecondaryObjects (cells)	Select input image: *Image generated by step 5*<br>Select the input objects: *Objects identified in step 3*<br>Select the method to identify the secondary object: Distance - B<br>Thresholding method: Adaptive Otsu<br>2 classes or 3 classes : 3 classes<br>Assign pixel in the middle : foreground<br>Threshold correction factor: 1.0<br>Lower and upper bounds on threshold 0.0001 – 1.0<br>Size of adaptive window: 100<br>Number of pixels by which to expand the primary objects? 10000
**7**	ConvertObjectstoImage	Select the input objects: *Objects identified in step 6*<br>Select the color format: Binary
**8**	MorphologicalSkeleton	Select input image: *Image generated by step 7*
**9**	MeasureObjectSkeleton	Select the seed object: *Objects identified in step 3*<br>Select the skeletonized image: *Image generated by step 8*<br>Maximum hole size: 25
**10**	ExporttoDatabase	

Supplementary Table 4

(Sub-) Entity	MYC(N) status	TP53 status	Cell line name	RRID[1]	PDT[2]	RA IC_{50} [µM]
NB	*MYCN* amp	mut (p.Cys135Phe)	SK-N-BE(2)-C	CVCL_0529	18 h	0.471
NB	*MYCN* amp	wt	NB-S-124	patient-derived model	n.a.	0.527
NB	*MYCN* amp	wt	CHP134	CVCL_1124	57.6 h	0.002
NB	no *MYC(N)* amp	wt	SH-SY5Y	CVCL_0019	>55 h	0.124
MB_{G3}	*MYC* amp	wt	HD-MB03	CVCL_S506	23.32 h	0.035
MB_{G3}	*MYC* amp	wt	Med8A	CVCL_M137	n.a.	0.659
MB_{G3}	*MYC* amp	mut (p.Val274Cys)	D425	CVCL_1275	61.92 h	0.001
MB_{SHH}	no *MYC(N)* amp	wt	ONS-76	CVCL_1624	18.6 h	> 100
$EPN_{ST\text{-}ZFTA}$	no *MYC(N)* amp	wt	DKFZ-EP1NS	n.a.	n.a.	89.74
$EPN_{PF\text{-}A}$	no *MYC(N)* amp	n.a.	EPD210FH	n.a.	n.a.	41.30
HGG_{wt}	*MYC* amp	mut (p.Arg273Cys)	SJ-GBM2	CVCL_M141	29 h	68.39
$pHGG_{wt}$	*MYCN* amp	wt	INF_R_1073r1_LTC	patient-derived model	n.a.	5.86
HGG_{G34R}	no *MYC(N)* amp	wt	INF_R_859p_LTC	patient-derived model	n.a.	3.54
$pHGG_{G34V}$	no *MYC(N)* amp	mut (p.Arg342Ter)	KNS-42	CVCL_0378	48 h	3.66
HGG_{K27M}	*MYCN* amp	mut (p.Lys132Arg)	SU-DIPG-13	CVCL_IT41	158.4 h	> 100
HGG_{K27M}	*MYC* amp	wt	SU-DIPG-25	CVCL_C1N0	n.a.	8.95

[1]RRID: Resource Identifier
[2]PDT: population doubling time as listed on Cellosaurus for the respective RRID
amp: amplification
wt: wild-type
n.a.: not available
LTC: longterm culture
